# Species Diversity of Lycoperdaceae (Agaricales) in Israel, with Some Insights into the Phylogenetic Structure of the Family

**DOI:** 10.3390/jof9101038

**Published:** 2023-10-22

**Authors:** Maxim Krakhmalnyi, Omoanghe S. Isikhuemhen, Mikael Jeppson, Solomon P. Wasser, Eviatar Nevo

**Affiliations:** 1 Department of Phytoecology, Institute for Evolutionary Ecology of National Academy of Sciences (NAS) of Ukraine, 37 Acad. Lebedev Str., 03143 Kyiv, Ukraine; 2 Institute of Evolution and Department of Evolutionary and Environmental Biology, Faculty of Natural Sciences, University of Haifa, 199 Abba Khousi Ave., Mt. Carmel, Haifa 3498838, Israel; spwasser@research.haifa.ac.il (S.P.W.); nevo@research.haifa.ac.il (E.N.); 3 Mushroom Biology and Fungal Biotechnology Laboratory, School of Agriculture and Environmental Sciences, North Carolina Agricultural and Technical State University, 207 Carver Hall, 1601 East Market Str., Greensboro, NC 27411, USA; omon@ncat.edu; 4 Department of Biological and Environmental Sciences, University of Göteborg, 40530 Göteborg, Sweden; jeppson@svampar.se; 5 N.G. Kholodny Institute of Botany of NAS of Ukraine, 2 Tereshchenkivska Str., 01601 Kyiv, Ukraine

**Keywords:** Agaricales, gasteroid fungi, taxonomy, molecular phylogeny, Israel

## Abstract

The diversity of Lycoperdaceae in Israel was studied. Molecular phylogenetic relationships within the family, and genus *Lycoperdon* in particular, were inferred using original ITS rDNA sequences of 58 samples belonging to 25 species from Israel and six other countries, together with 66 sequences stored in the GenBank database. The current molecular phylogenetic study recovered the family Lycoperdaceae as a monophyletic group, which was supported in both maximum likelihood and Bayesian analyses. The clades corresponding to the genera *Apioperdon*, *Bovista*, *Calvatia*, *Disciseda*, and *Lycoperdon* were revealed. The taxonomic structure of the named genera was partially resolved. Within the genus *Lycoperdon*, some species received significant statistical support; however, their relationships, as well as the problem of the genus monophyly, mostly remained questionable. As a result of a thorough literature survey, extensive sample collection, and studies of the material stored in the Herbarium of the Institute of Evolution, University of Haifa (HAI, Israel), fifteen species representing five genera were found in the territory of Israel. Six species, namely *Apioperdon pyriforme*, *Bovista aestivalis*, *Calvatia candida*, *Lycoperdon decipiens*, *L. niveum*, and *L. perlatum*, are new additions to the diversity of Lycoperdaceae in Israel. Detailed macro- and micromorphological descriptions, ecology, geography, and critical notes, together with light microscopy photos and SEM micrographs, are provided. In-depth discussion on some taxonomically challenging species is presented.

## 1. Introduction

The family Lycoperdaceae Chevall. comprises species of Agaricales Underw. with an enclosed (angiocarpic) fruit body development and lack of a true stipe, incorporating so-called “true puffballs”—classic gasteroid genera *Lycoperdon* Pers., *Bovista* Pers., *Calvatia* Fr., *Disciseda* Czern., together with some recent taxonomic additions. The size of the representatives of the family varies tremendously, from 3–9 (2–15) mm gasterocarps of *Bryoperdon acuminatum* (Bosc) Vizzini (=*Bovista acuminata* (Bosc) Kreisel) [1,2,3,4], arguably the smallest puffball, to the gigantic fruit bodies of *Calvatia gigantea* (Batsch) Lloyd (=*Langermannia gigantea* (Batsch) Rostk.), reaching more than one meter in diameter and weighing up to 20 kg [5]. The shape of the gasterocarps could be globose, subglobose, turbinate, sessile, pyriform, pestle-shape, etc. The spore-forming inner tissue, gleba, is whitish at first, turning to olivaceous, brown, or purplish-colored powdery mass, comprised of mature basidiospores. The gleba is covered with a protective sterile peridium, which usually can be segregated into the exoperidium (the outer layer) and endoperidium (the inner layer). The former is typically ornamented with spines, warts, and granules, and disintegrates after the gasterocarp’s maturation, revealing the endoperidium, which either has a more or less regular apical opening, called an ostiole, or just irregularly ruptures and disintegrates, setting free powder containing billions or even trillions of basidiospores [6]. Later, the spores are passively dispersed by environmental factors such as wind, raindrops, insects, etc. The family has a cosmopolitan distribution with its members found in temperate, arid, and tropical climates [5]. Members of Lycoperdaceae, along with other representatives of the former “Gasteromycetes”, have a rich folklore heritage and have been used in folk medicine by different cultures around the world. Burk presented a comprehensive literature review on the usage of puffballs by North American Indians [7]. The spectrum of their applications included religious, decorative, hemostatic, dietary, and other purposes. Some members of the family were studied for medicinal properties. Calvacin, calvatan, craniformin, and rubroflavin, all isolated from representatives of the genus *Calvatia*, demonstrated anti-tumor activity, based on immunopotentiation [8,9]. Altuner et al. observed antimicrobial effects of the ethanolic extract of *Bovista nigrescens* Pers. against several Gram-positive and Gram-negative microorganisms, especially *Bacillus subtilis*, *Klebsiella pneumonia*, and *Staphylococcus carnosus* [10].

Counting the number of Lycoperdaceae members presents a challenging issue. This is largely due to the lack of a world monograph devoted to the family (this situation also holds true for many other gasteroid taxa). In addition, a considerable number of unresolved taxonomic questions regarding genera structure, synonyms, and even the place of the family within the modern Agaricales further complicates this task. Kirk et al. reported approximately 150 species as members [11]. This figure obviously does not represent the real number of species known to date, because many novel species have been recently described [12,13,14,15,16,17,18,19,20,21,22,23,24]. The Encyclopædia Britannica web resource lists 160 species [25], but this number includes the “earthstars”—representatives of the genus *Geastrum* Pers. Incorporation of puffballs and earthstars into a single family reflects a fairly common taxonomic concept of the past. Krüger et al. demonstrated that the earthstars represent a separate lineage and have evolved independently of Lycoperdales [26]. The family Geastraceae Corda is currently placed in the subclass Phallomycetidae K. Hosaka, Castellano & Spatafora, proposed by Hosaka et al. [27] on the basis of molecular phylogenetic studies. In more recent additions to the Dictionary of the Fungi [28], the family Lycoperdaceae is treated as a synonym of Agaricaceae Chevall.

Lycoperdaceae has been traditionally placed in the order Lycoperdales [29,30], which contained earthstars, puffballs, and their allies. Lycoperdales was one of the major orders within “Gasteromycetes” [29,31,32]—an assemblage of taxa, which was characterized by spores maturing inside enclosed fruit bodies and dispersed by environmental factors, due to loss of ballistospori. An evolutionary approach, applied to taxonomical classification of various gasteroid morphologies, as well as numerous molecular phylogenetic studies [27,33,34,35,36,37], proved that the group represents an artificial assemblage of polyphyletic taxa belonging to different lineages. All the taxa from the “Gasteromycetes” were transferred to the class Agaricomycetes Doweld. As for the family Lycoperdaceae, it is either left intact, preserving its rank, or is incorporated into Agaricaceae sensu lato, which already consumed a number of families of the former “Gasteromycetes”, e.g., Battarreaceae, Montagneaceae, Mycenastraceae, Podaxaceae, and Tulostomataceae. The latter view is supported by several studies showing that Lycoperdaceae is nested as a separate clade within the Agaricaceae. Parsimony analysis of nuc-SSU datasets, including sequences of some representatives of Agaricaceae, Lycoperdaceae, Geastraceae, and Phallaceae, demonstrated that the puffballs were nested within the euagarics clade, which received a bootstrap support of 78% [26]. Intriguingly, *Lepiota cristata* (Bolton) P. Kumm. was found to be genetically closer to *Mycenastrum corium* (Guers.) Desv. and the true puffballs, than to *Macrolepiota procera* (Scop.) Singer and *Macrolepiota konradii* (Huijsman ex P.D. Orton) M.M. Moser. Members of Lycoperdaceae grouped in a clade with 72% bootstrap support. Judging from these results, Lycoperdaceae was considered closely related to Lepiotaceae, and the authors suggested the inclusion of the order Lycoperdales in the Agaricales [26].

The analysis conducted by Vellinga, which involved 160 specimens, including some representatives of the genera *Bovista*, *Calvatia*, and *Lycoperdon*, revealed that these species form an independent “Lycoperdaceae” clade within the Agaricaceae [38]. However, the resulting trees, derived from individual ITS and nuc-LSU, and combined ITS + LSU datasets, presented different topologies; for instance, in the LSU phylogram, six taxa of Lycoperdaceae formed a distinct clade closely related to the agaricoid genera *Agaricus* L., *Chlorophyllum* Massee, *Leucoagaricus* Locq. ex Singer, and *Leucocoprinus* Pat., while in the combined ITS + LSU phylogram, a single taxon representing Lycoperdaceae—*L. pyriforme* (= *Apioperdon pyriforme*) took a basal position in respect to the core Agaricaceae clade.

Thus, Lycoperdaceae could be treated as a gasteroid lineage within Agaricaceae, losing its family rank [33,39,40]. In this case, a broader concept of Agaricaceae is necessary, which, on the other hand, will inevitably lead to the situation in which we will have a family that unites taxa with rather discrepant anatomical characters, while looking artificial from the point of classical taxonomy. The opposite idea is to postpone the final decision regarding the taxonomic status of the family Lycoperdaceae until more advanced phylogenetic reconstructions of Agaricaceae and Agaricales, in general, will be established [41].

Difficulties also occurred when scientists tried to resolve the phylogenetic relations within Lycoperdaceae itself. One can see a somewhat “foggy” concept of genera in the family. Different mycologists had their personal views and opinions regarding the taxonomical composition of Lycoperdaceae, genera richness, and major distinctive characteristics, which would reliably delimit the genera from one another and allow the proper assignment of species. For instance, in the case of *Vascellum pratense* (Pers.) Kreisel, which is registered for Israel, some researchers believed that the presence of a morphological feature, such as a diaphragm, was sufficient to place the species in the genus *Vascellum* F. Šmarda [5,42]. However, this proved to be unsubstantiated from a molecular perspective: phylogenetic studies [2,21,41,43,44] showed that *V. pratense* groups with core species of the genus *Lycoperdon*, including *Lycoperdon perlatum* Pers.: Pers., *L. marginatum* Vittad. and *L. norvegicum* Demoulin, and should be treated as *Lycoperdon pratense* Pers.

Since the beginning of the 21st century, the family Lycoperdaceae and its members have been the subject of numerous molecular phylogenetic studies [14,15,18,26,40,41,43,45,46,47]. Listed works presented phylogenetic reconstructions, which were based on the following gene datasets: ITS rDNA, nuc-LSU, nuc-SSU, mt-LSU, and mt-SSU rDNA. Larsson and Jeppson provided one of the most comprehensive molecular phylogenetic examinations of the family Lycoperdaceae [41]. The authors used a combined ITS and LSU rDNA dataset, and sampled 79 specimens belonging to 47 species, which were mostly of European origin, along with some sequences selected from the GenBank database. Their analysis demonstrated that Lycoperdaceae is a monophyletic group, containing four major clades—*Lycoperdon*, *Calvatia*, *Bovista*, and *Disciseda*, more or less corresponding to the equally named genera. The structure and boundaries of the genus *Lycoperdon*, the type genus of the family, presented in the phylograms, are also of great interest. A number of species that were traditionally placed in the related genera were nested within the “*Lycoperdon*” clade, including *Bovista dermoxantha* (Vittad.) De Toni, *Bovistella radicata* Pat., *Calvatia cretacea* (Berk.) Lloyd, *C. turneri* (Ellis & Everh.) Demoulin & M. Lange, *Handkea excipuliformis* (Scop.) Kreisel, *H. utriformis* (Bull.) Kreisel, and *Morganella fuliginea* (Berk. & M.A. Curtis) Kreisel & Dring. The “*Lycoperdon*” clade splits into two subclades. The first subclade contained the type species of the genus, *Lycoperdon perlatum*, which grouped with representatives of the genera *Morganella* Zeller and *Vascellum*, as well as with two other members of *Lycoperdon*. The second subclade absorbed the main diversity of *Lycoperdon* species, together with some taxa assigned to *Bovista*, *Bovistella*, *Calvatia*, and *Handkea* Kreisel, which have already been mentioned above. Larsson and Jeppson [41] chose to accept a broad interpretation of *Lycoperdon* and proposed to recognize the subgenera *Vascellum*, *Morganella*, *Bovistella*, *Utraria*, and *Apioperdon*, in addition to *Lycoperdon* s. str. The authors noted that allocation of some species remained questionable and suggested subdivision was conditional to some extent.

Vizzini and Ercole [2] conducted a study that aimed to detect the phylogenetic position of *Bovista acuminata*, a very small puffball, characterized by oblong, conical basidiomes, lack of subgleba, “*Lycoperdon*” type capillitium with small pores, globose, almost smooth to finely verrucose spores, and bryophilous association. In the analysis [2], both *B. acuminata* sequences (collections from Italy) clustered in a well-supported clade (BPP—1.00) outside *Lycoperdon*. The clade was sister to *L. pyriforme*. Based on morphological, ecological, and molecular data, Vizzini and Ercole erected a new genus *Bryoperdon* Vizzini, creating a new combination—*Bryoperdon acuminatum* (Bosc) Vizzini [2]. In the same paper, the authors also raised a question regarding the taxonomic placement of *L. pyriforme*, whose isolated position had already been pointed out in a number of molecular phylogenetic studies [40,41,43,46]. Vizzini and Ercole proposed to elevate the rank of *Apioperdon*, considering it a distinct genus within Lycoperdaceae [2].

Alfredo et al. [14] focused their efforts on the reevaluation of Brazilian species described under *Morganella*, and examined seventy specimens from Brazilian, NY and PDD herbaria. Analyses of combined ITS and LSU datasets demonstrated that all specimens designated as *Morganella* (except for *M. sulcatostoma* C.R. Alves & Cortez) grouped together in a single clade (84% BS and 0.9 BPP) corresponding to the subgenus *Morganella* within *Lycoperdon*. The absence of capillitium and reduced, compact cellular subgleba were defined as the most important features of the subgenus. One new species, *Lycoperdon oblongatum* Accioly, Baseia & M.P. Martín, and six new combinations were proposed. Additionally, the authors questioned the validity of nomenclatural changes introduced by Vizzini and Ercole [2], citing the small number of specimens involved in the mentioned analysis.

The study of the family Lycoperdaceae in Israel began with the work of Reichert and Avizohar-Hershenzon, who published an article on higher fungi of Israel, where they reported on *Disciseda cervina* (Berk.) Hollós, found near Rehovot (Philistean Plain) [48]. The authors gave a very brief description of macro- and micromorphological characteristics of studied specimens, together with black and white photos of their sporocarps. In 1963, Dring and Rayss published a paper focused exclusively on the diversity of gasteroid fungi in Israel, which was unique for that stage of Israeli mycobiota studies [49]. As a result, twenty-eight species and intraspecific taxa were new records for the territory of the country, including a novel species for science—*Scleroderma multiloculare* Dring & Rayss. The article provided a description of the family Lycoperdaceae and four genera, two of which—*Lycoperdon* and *Bovista*—were new for Israeli mycobiota. Within these genera, six species were counted—*Lycoperdon pratense* (as *Vascellum pratense*), *Lycoperdon lividum* Pers. (as *Lycoperdon spadiceum* Pers.), *Bovista pusilla* (Batsch) Pers. (as *Lycoperdon pusillum* Batsch ex Schumacher), *Bovista plumbea* Pers., *Bovista nigrescens* Pers., and *Disciseda bovista* (Klotzsch) Henn. Each species was provided with a short description, origin of specimen, pictures of spore and capillitium morphology (some species had photos of gasterocarps), and critical notes. It should be noted that in the same study, Dring and Rayss questioned previous records of *D. cervina*, and also mentioned the genus *Mycenastrum* Desv., reporting the presence of *Mycenastrum corium* in Israel [49]. The authors placed the species in Lycoperdaceae, which was typical for that period. Molecular phylogenetic analyses of Krüger et al. [26] and Larsson and Jeppson [41] showed that Lycoperdaceae could remain monophyletic only with the exclusion of *M. corium*.

Twenty years later, Binyamini, who made a great contribution to the knowledge of fungi in Israel, and gasteroid fungi in particular [50,51,52,53,54], published a book [55] in which he counted several species of Lycoperdaceae that were not known for Israel from the previous studies. These were *Lycoperdon molle* Pers., *Lycoperdon atropurpureum* Vittad., and *Calvatia gigantea* (Batsch) Lloyd (as *Lycoperdon giganteum* Batsch). Binyamini presented photos of gasterocarps of mentioned species, but he gave only brief descriptions, without any drawings or photos revealing micro-characteristics (spores, capillitium, etc.). The author also did not supply data on the origin of the studied specimens. Unfortunately, the Herbarium of Binyamini, stored in Tel-Aviv University (TELA), has been closed for many years; therefore, the specimens of Lycoperdaceae, collected and identified by Binyamini, were not available for the current study.

Krakhmalnyi presented his PhD thesis on the gasteroid basidiomycetes of Israel, where he gave a thorough treatment of the group, including the family Lycoperdaceae [56]. Yet, some information that was provided in the study is currently outdated.

## 2. Materials and Methods

### 2.1. General

This study is based on the investigation of material stored in the Herbarium of the Institute of Evolution, University of Haifa (HAI, Israel) and the University of Liege Herbarium (LG), and on new fungal samples collected during expeditions to different botanical–geographical regions of Israel from October 2011 to March 2013.

During sample collection, the following information was recorded: the possible field identification of the specimen, locality, date of collection, collector’s name, and additional information (relevant descriptive notes, nearby vegetation, etc.). For color interpretation “Flora of British Fungi: Colour Identification Chart” was used [57]. The photos of gasterocarps were taken with an Olympus EPL-1 digital camera. Collected samples were dried at 50 °C, and then transferred to separate polyethylene zip bags for storage.

The information pertaining to general distribution and habitat of examined species was obtained and generalized using multiple sources, including online databases: The Global Biodiversity Information Facility (GBIF) [58], Species 2000 & ITIS Catalogue of Life (http://www.catalogueoflife.org), The Atlas of Living Australia (Atlas) (http://www.ala.org.au/), Harvard University Herbaria and Libraries (http://huh.harvard.edu/); and checklists dealing with gasteroid fungi, monographs, identification guides, and other literature sources [1,3,5,29,55,59,60,61,62,63,64,65]. Nomenclature was checked and analyzed using multiple publications, in addition to the Index Fungorum (http://www.indexfungorum.org) and MycoBank (http://www.mycobank.org/) online databases.

**Figure 1:**
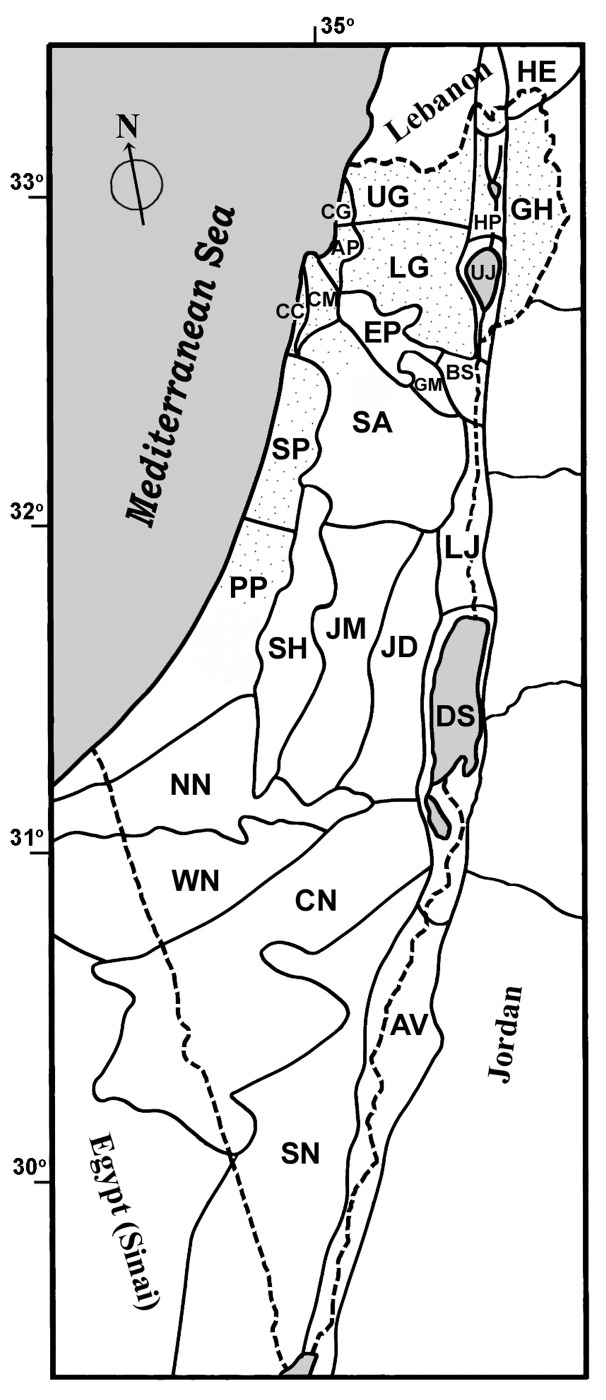
Accepted abbreviations of nature regions of Israel: AP—Akko Plain; AV—Arava Valley; BS—Beit Shean Valley; CC—Carmel Coast; CG—Coast Galilee; CM—Carmel Mount; CN—Central Negev; DS—Dead Sea Area; EP—Esdraelon (Yizre’el) Plain; GH—Golan Heights; GM—Gilboa Mount; HE—Hermon Mount; HP—Hula Plain; JD—Judean Desert; JM—Judean Mts.; LG—Lower Galilee; LJ—Lower Jordan Valley; NN—Northern Negev; PP—Philistean Plain; SA—Samaria; SH—Shefela; SN—South Negev; SP—Sharon Plain; UG—Upper Galilee; UJ—Upper Jordan Valley; WN—Western Negev.

Distribution of species in Israel is based on phytogeographic districts (Figure 1) proposed by Feinbrun-Dothan and Danin [66].

### 2.2. Microscopy Studies

Some characteristics of studied specimens (structure of exo- and endoperidium, gleba and subgleba, etc.) were examined under a ZEISS Stemi DV4 stereo microscope (Carl Zeiss AG, Oberkochen, Germany), and pictures were taken with a Canon Power Shot G10 digital camera. Micromorphology, including spore and capillitium hyphae structure, was observed under ZEISS Axiostar 1122–100 (Carl Zeiss AG, Oberkochen, Germany) and Olympus BX51 (Olympus LS, Tokyo, Japan) light microscopes. Measurements were made using an UPlanFLN 100x/1.30 (Oil) immersion objective, and ≥30 spores of each specimen were measured for the statistical calculation. Melzer’s reagent, 3% aqueous KOH, and Lactophenol Cotton Blue were used as mounting media. Spore size is given as follows: (min) average minus standard deviation—average plus standard deviation (max). Microscopic photos were taken with Canon Power Shot G10 and Canon EOS 5D Mark III digital cameras.

Specimens were also studied under a field emission scanning electron microscope (FE-SEM) Mira 3 LMU (TESCAN, Brno, Czech Republic) with integrated Oxford instruments INCA energy (Oxford instruments, Abingdon, UK), and the following scanning electron microscopes (SEMs): Philips SEM 515 (Philips Electronics N.V., Eindhoven, The Netherlands) and JSM-6060 LA (JEOL, Ltd., Tokyo, Japan). A small dried gleba sample with spores of each specimen was mounted on a metal stub using electroconductive glue, then coated with a thin layer of gold in fine coat ion sputter JFC-1100 (JEOL, Ltd., Tokyo, Japan), and subsequently analyzed using FE-SEM and SEM.

### 2.3. Taxon Sampling for the Phylogenetic Study

The phylogenetic relations between species and genera within the puffball family Lycoperdaceae were investigated using molecular methods, in conjunction with the examination of macro- and micromorphological characteristics, to re-evaluate the systematic position of some controversial species, resolve the taxonomic structure of the family, and reinforce the identification of Israeli material. Fifty-eight samples from Israel and other countries (Belgium, France, Germany, Luxembourg, Ukraine, USA), comprising twenty-five species, which belong to *Apioperdon*, *Bovista*, *Calvatia*, and *Lycoperdon* genera, deposited in the Herbarium of the Institute of Evolution, University of Haifa (HAI) and the University of Liege Herbarium (LG), together with sixty-six previously published sequences from GenBank database (NCBI, https://www.ncbi.nlm.nih.gov/genbank/), including specimens assigned to *Bovista*, *Bovistella*, *Calvatia*, *Disciseda*, *Handkea*, *Holocotylon*, *Lepiota*, *Lycoperdon*, *Morganella*, *Mycenastrum*, *Tulostoma*, and *Vascellum* were the subject of the current phylogenetic analysis. Information about the specimens and GenBank accession numbers are provided in Table 1.

### 2.4. DNA Extraction

Approximately 30 to 100 mg of glebal tissue from dried herbarium material or fresh samples were placed in 1.5 mL Eppendorf tubes. Two sterile metal beads were added to each tube. The tubes were subsequently submerged in liquid nitrogen for 5–10 s, and immediately after that homogenization of material was carried out using a Qiagen Tissue Lyser II (Retsch, Haan*,* Germany) for 3 min at 30 Hz. After homogenization, CTAB and SDS (10%) extraction buffers were added directly onto the crushed material, then Eppendorf tubes were incubated in a water bath for 12 h at 65 °C. In the extraction DNA enrichment procedure, phenol:chloroform:isoamyl alcohol 25:24:1 (Sigma-Aldrich Co., St. Louis, Missouri, USA) was used first, and chloroform-isoamyl alcohol 24:1 (SEVAG) solution thereafter. Isolated DNA was cleaned by washing with ≈100% isopropanol (Sigma-Aldrich Co.) and 70% ethanol (Sigma-Aldrich Co.), and then dried. The dried DNA was diluted in 50 µL 0.1 M TE buffer and 1 µL of RNase A (1 mg/mL) (Sigma-Aldrich Co.) was added. The concentration of obtained DNA was measured with a NanoDrop^®^ ND-1000 spectrophotometer (NanoDrop Technologies, Montchanin, DE, USA) and adjusted to an average value of 20 ng/μL for downstream applications.

### 2.5. PCR Amplification, Purification, Sequencing

Nuclear ribosomal internal transcribed spacer (ITS) was amplified. The amplification of the whole ITS region (ITS1–5.8S–ITS2) of rDNA was carried out using primers ITS1F and ITS4B (Sigma-Aldrich Co.) [70]. The polymerase chain reaction (PCR) was mixed in a total volume of 15 μL containing: 7.5 µL of Reddy Mix (PCR Master Mix, Thermo Fisher Scientific, Waltham, Massachusetts, USA), 0.4 µL of each primer, 3.7 µL of molecular water, and 3 μL of genomic DNA (with the usual quantity of 60 ng/per reaction). The PCR amplification was carried out on a GeneAmp^®^ PCR System 9700 (Applied Biosystems, Inc., Waltham, Massachusetts, USA). The basic PCR cycling conditions were as follows: an initial denaturation step of 95 °C for 5 min, followed by 35 cycles of denaturation at 95 °C for 30 s, primer annealing at 55 °C for 30 s, primer extension at 72 °C for 1 min, and a final elongation step at 72 °C for 7 min. If samples were not responding to this program, some parameters (number of cycles, annealing temperature, quantity of DNA per reaction) were individually adjusted for better output results.

All PCR products were visualized in 1% (*w*/*v*) agarose gels, stained with GelRed (Nucleic acid stain, Biotium, Fremont, CA, USA) and viewed under ultra-violet light on a Molecular Imager Gel Doc XR with BioRAD Quantity One 4.5.2 Software. Sizes of PCR amplicons were estimated against a GeneRuler 100 bp DNA Ladder (Thermo Fisher Scientific). Prior to DNA sequencing, PCR products were cleaned using an illustra ExoProStar 1-Step (GE HealthCare, Chicago, Illinois, USA) following the manufacturer’s instructions.

Cleaned PCR products were sequenced using a Big Dye Terminator V1.1 Cycle Sequencing Kit (ABI, Austin, TX, USA) in two different reactions with ITS1F and ITS4B primers. They were subsequently analyzed on an Applied Biosystems (AB) 3130xl Genetic Analyzer (Hitachi, Ltd., Tokyo, Japan).

### 2.6. Phylogenetic Analysis

The whole ITS rDNA region dataset comprised 124 sequences, 58 of which were obtained during the current research and 66 were acquired from the GenBank database. Sequences were aligned individually for each locus using the MUSCLE software package in Mesquite 2.73 [71], and the alignment was inspected and adjusted manually with ambiguous regions excluded. Total length of the alignment was 704 bp. After exclusion of ambiguous regions, the length of the alignment decreased to 639 bp. Phylogenetic relationships within the family Lycoperdaceae were determined by maximum likelihood (ML) and Bayesian Interference (BI) methods. Phylogenetic support was assessed via the bootstrap method using PAUP* v.4.0a109 [72], and posterior probability for ML and BI analyses, respectively. The software Garli-1.0 [73] for ML and MrBayes 3.1.2 [74] for BI methods on the CIPRES Science Gateway V. 3.1 (www.phylo.org) were used. Analyses were conducted applying the GTR + C + I substitution model and rate heterogeneity with unlinked parameters. One thousand bootstrap replicates were computed for ML. For Bayesian phylogenetic estimations, parallel runs of four chains were computed to 20 million generations, with sampling every 1000 generations. Trees were sampled when an equivalent posterior probability plateau was achieved between runs. Statistical support was recognized as significant with ˃70% bootstrap values (BS) for ML, and ˃0.95 posterior probabilities (BPP) for BI. *Tulostoma kotlabae* and *Lepiota cristata* were selected as the outgroup. *Mycenastrum corium* was also included in the analysis.

## 3. Results and Discussion

As a result of a thorough literature survey, extensive collections of fresh samples, and examination of material stored in the HAI herbarium, fifteen species belonging to five genera were found in the territory of Israel (Table 2). Six species, including *Apioperdon pyriforme* (Schaeff.) Vizzini, *Bovista aestivalis* (Bonord.) Demoulin, *Calvatia candida* (Rostk.) Hollós, *Lycoperdon decipiens* Durieu & Mont., *L. niveum* Kreisel, and *L. perlatum* Pers.: Pers. are new additions to the diversity of the family Lycoperdaceae and the whole gasteroid mycobiota in Israel.

### 3.1. Molecular Phylogeny of Lycoperdaceae

**Figure 2:**
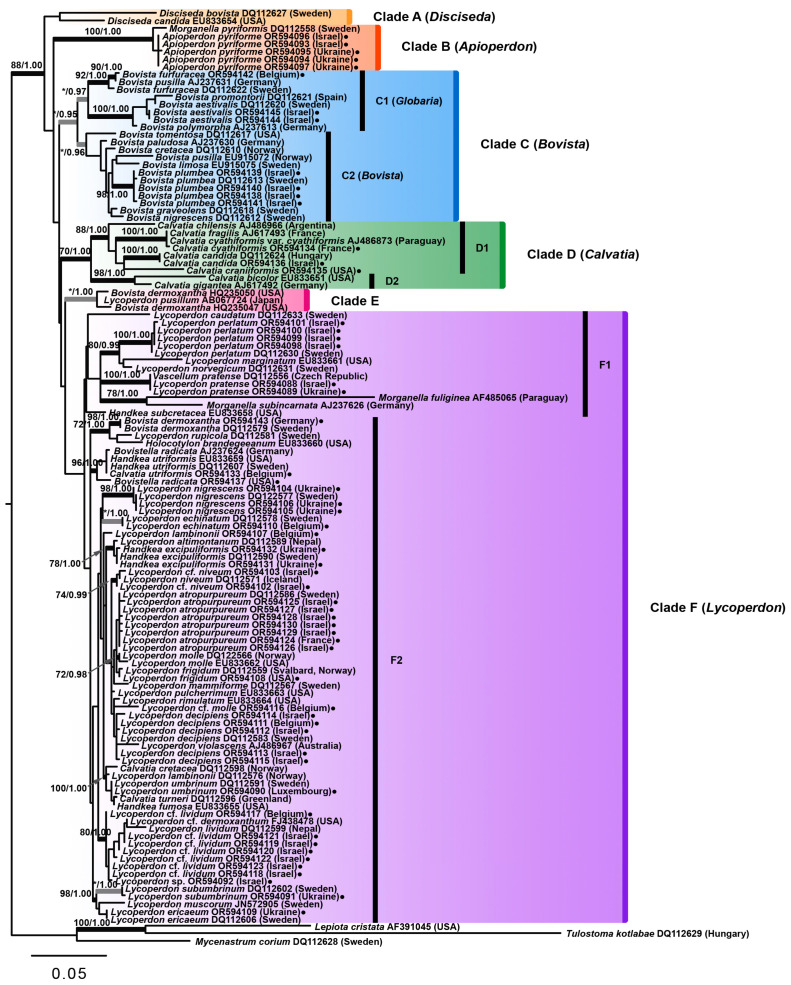
Maximum likelihood tree of the family Lycoperdaceae based on the whole ITS sequence dataset. The topology of the Bayesian tree, in general, was congruent with the ML tree, but had more supported branches. Thick black lines indicate branches with significant statistical support (>70% bootstrap value for ML and >0.95 posterior probability for Bayesian analysis). Thick grey lines indicate branches significantly supported only by the Bayesian method. Respective BS and BPP values are given near the branches. All sequences are provided with full taxonomic names, GenBank accession number, and country of origin. The sequences obtained during the current study are marked with a dot.

Of all the tested algorithms, the maximum likelihood tree of the family Lycoperdaceae had the highest number of supported clades, and the Bayesian tree showed, in general, a similar topology (Figure 2). In the current study, Lycoperdaceae was recovered as a monophyletic group with significant statistical support in both ML and Bayesian analyses (88% BS and 1.00 BPP). The outgroup was comprised of *T. kotlabae* together with *L. cristata*. The position of *M. corium* relative to the members of the family is still an open question. The monotypic genus *Mycenastrum* for a long time had been placed in Lycoperdaceae, although Zeller [75] considered that distinctive macro- and micromorphological characteristics, including large subglobose to irregularly shaped gasterocarps, a thick endoperidium, the lack of an ostiole, pitted basidiospores, and thick-walled, elastic, non-poroid, non-septate capillitial threads with spinose branches were sufficient to establish a separate family, i.e., Mycenastraceae. In the study of Bates et al., *M. corium* was treated as a basal member of Lycoperdaceae, although the authors expressed their awareness that in terms of its unique morphological features, the species is clearly separated from other representatives of the family [43]. The current study recovered *M. corium* as a sister taxon to the rest of the species composing the Lycoperdaceae ingroup, which agrees with results presented by Krüger et al. [26] and Larsson and Jeppson [41]. The question of *M. corium* assignment either to Lycoperdaceae or Mycenastraceae becomes even more entangled and somewhat incorrect in the situation when both families tend to be incorporated into the Agaricaceae s.l., which will inevitably lead to the reduction in their taxonomic rank.

Within Lycoperdaceae, six main clades were revealed. Clade A contained two species of the genus *Disciseda*—*D. bovista* (GenBank no. DQ112627, Sweden) and *D. candida* (GenBank no. EU833654, USA), and represented an unsupported sister clade to the remaining species of the family. The genus *Disciseda* has a worldwide distribution and is characterized by the exoperidium persisting as a disc at the top of the gasterocarp, a basal position of the mouth, and a peculiar inversion of mature gasterocarps [3].

Clade B comprised six sequences of *Apioperdon pyriforme* representing the material from Israel (OR594093, OR594096), Ukraine (OR594094, OR594095, OR594097), and one sequence obtained from the Swedish specimen by Larsson and Jeppson [41]. The clade had significant statistical support (100% BS and 1.00 BPP) and was located more basally within the family, supporting the data of Bates et al. [43] and Kim et al. [21], which, however, do not agree with the topologies presented in a number of studies [2,14,41,44]. The sequences obtained from Ukrainian and Israeli specimens clustered together, while the sequence GenBank no. DQ112558 (Sweden) occupied the sister branch. BLAST analysis showed that the sequence of *A. pyriforme* OR594096 is 99% similar to the sequence of *Morganella pyriformis* GenBank no. DQ112557 [41], having one base insertion, one base deletion, and four substitutions difference, while the sequence of *L. pyriforme* GenBank no. AY854075 demonstrated the closest match, with the difference in just two substitutions.

Clade B was a sister group to the remaining species within Lycoperdaceae. The basal position of *A. pyriforme*, observed both in the present analysis and in the analysis of Bates et al. [43], was neither supported by ML nor by Bayesian methods. Although *A. pyriforme* was incorporated into the lignicolous genus *Morganella* by Krüger and Kreisel on the basis of molecular data and ecological features [45], in the current study it did not cluster with sampled representatives of the genus—*M. fuliginea* (AF485065) and *M. subincarnata* (AJ237626). The same picture was observed in the investigations of Krüger et al. [26], Larsson and Jeppson [41], Bates et al. [43], and Alfredo et al. [14]. *Apioperdon pyriforme* is similar to members of *Lycoperdon* in having a true capillitium (which is, however, not pitted), and to the genus *Morganella* by its lignicolous habitat. At the same time, it differs from both by possessing a distinctly white cellular subgleba and by the structure of exoperidium sphaerocysts. Based on these distinctive characteristics, Krüger and Kreisel proposed a new subgenus *Apioperdon* Kreisel et D. Krüger within the genus *Morganella*, to accommodate *M. pyriformis* [45]. In the analysis of Larsson and Jeppson, *A. pyriforme* occupied a basal position relative to the genus *Lycoperdon*, and the authors chose to distinguish *Apioperdon* (Kreisel & D. Krüger) Jeppson & E. Larss. as a monotypic subgenus within *Lycoperdon* [41]. Later, Vizzini and Ercole made a decision to elevate the position of *Apioperdon* to the genus level, based on cumulative morphological, ecological, and molecular evidence, and proposed a new combination—*Apioperdon pyriforme* (Schaeff.) Vizzini [2]. Our analysis solidifies the status of *Apioperdon* as a separate genus within Lycoperdaceae.

Clade C represented the genus *Bovista* in the current analysis and was supported only by the Bayesian method (0.95 BPP). The clade splits into two subclades, C1 and C2, corresponding to the subgenera *Globaria* and *Bovista*, respectively. The *Globaria* subclade united species, which are characterized by intermediate to “*Lycoperdon*” types of capillitium, whereas the *Bovista* subclade incorporated species with dichotomously branched capillitium and pedicelate spores. Neither of the subclades had significant ML support, but both “*Globaria*” and “*Bovista*” received Bayesian support of 0.97 BPP and 0.96 BPP, respectively. The phylograms presented by Larsson and Jeppson showed a similar topology with the same species composition of each subclade [41]. However, in their analysis, the subclades *Globaria* and *Bovista* were well supported by both maximum parsimony and Bayesian methods, while the whole *Bovista* clade had only Bayesian support. The *Globaria* subclade in the current study was represented by *B. aestivalis*, *B. furfuracea*, *B. polymorpha*, and *B. promontorii*. Sequences of two Israeli specimens of *B. aestivalis* (OR594144 and OR594145) clustered together with the Swedish specimen (GenBank no. DQ112620), along with two sequences assigned to *B. promontorii* (GenBank no. DQ112621) and *B. polymorpha* (GenBank no. AJ237613), in a well-supported clade. BLAST analysis showed that the sequence of *B. aestivalis* OR594145 is 99% similar to the sequence of *B. aestivalis* (GenBank no. EU833650) from the study of Bates et al. [43], with a difference in four substitutions, and is also 99% similar to the mentioned sequence of *B. aestivalis* GenBank no. DQ112620 [41], with a difference in two deletions (one base pair each) and one base substitution.

The sequence of *B. pusilla* (GenBank no. AJ237631) from the study of Krüger et al. [26], which also nested within the *Globaria* subclade, clustered with two sequences of *B. furfuracea* (specimens from Belgium and Sweden) and, more likely, represents the same species. Larsson and Jeppson [41] noted that Krüger et al. [26] probably used Kreisel’s concept of *B. pusilla* when identifying the specimen.

The *Bovista* subclade (C2) accommodated species with “*Bovista*” type capillitium and pedicellate spores. Apart from the type species of the genus, *B. plumbea*, the subclade included *B. cretacea*, *B. graveolens*, *B. limosa*, *B. nigrescens*, *B. paludosa*, *B. pusilla*, and *B. tomentosa*. Four sequences of *B. plumbea* acquired from Israeli material clustered with the sequence of *B. plumbea* (GenBank no. DQ112613) originated from the Swedish sample in a clade with significant statistical support (98% BS/1.00 BPP).

Clade D accommodated members of the genus *Calvatia*, including *C. gigantea* (= *Langermannia gigantea*). Main characters of the genus are irregular rupturing of the peridium and medium- to large-sized gasterocarps. The clade received significant support according to both ML (70% BS) and BI methods (1.00 BPP). The topology of the clade within Lycoperdaceae suggested a close relationship with the genus *Lycoperdon*. The same picture was observed in the phylogenetic reconstructions of Bates et al. [43]. However, this position of the *Calvatia* clade did not receive significant statistical support in either analysis. Phylogenetic trees presented in the studies of Larsson and Jeppson [41] and Alfredo et al. [14] showed a more basal position of the genus *Calvatia*. The *Calvatia* clade splits into subclades D1 and D2, both receiving significant ML and Bayesian support—88% BS/1.00 BPP and 98% BS/1.00 BPP, respectively (Figure 2). The subclade D1 contained sequences belonging to *C. candida*, *C. chilensis*, *C. craniiformis*, *C. cyathiformis*, and *C. fragilis*. BLAST analysis showed that the sequence of *C. candida* obtained from Israeli material (OR594136) is 99% similar to the sequence of *C. candida* presented in the study of Larsson and Jeppson [41] GenBank no. DQ112624, with the difference in only one base marked as “N” in the latter sequence. These two sequences of *C. candida* acquired from Israeli and Hungarian specimens clustered in a well-supported clade. The final subclade within D1 that received significant support from BS and BI analyses was comprised of two *C. cyathiformis* sequences—one obtained during the current study (OR594134, Corse, France) and the other GenBank sequence no. AJ486873, along with the third sequence (GenBank no. AJ617493), which was deposited in the GenBank database under the name “*C. fragilis*”. There is no clear understanding whether *C. cyathiformis* and *C. fragilis* are two separate species. Some mycologists considered the latter as a form of *C. cyathiformis* [76], and molecular data support their close relationship. However, there is a number of macro- and micromorphological characteristics on the basis of which these species can be delimited. For instance, Bates et al. observed a significant difference in spore ultrastructure between the specimens of *C. cyathiformis* and *C. fragilis* under SEM [43]. Thus, the question of their demarcation remains open.

The subclade D2 contained two sequences belonging to *C. gigantea* and *C. bicolor*. These two species also clustered together in the phylogram presented by Bates et al. [43], although their position in relation to other species of the genus *Calvatia* was not statistically supported. In the study of Kim et al., *C. gigantea*, *C. bicolor* and “*C. pachydermica*” (*C. pachyderma*, voucher AN014692) formed a well-supported clade (95% BS / 1.0 BPP) [21]. In the current analysis, the subclade D2 received support by both ML and BI methods (98% BS and 1.00 BPP). Larsson and Jeppson suggested retaining the wide concept of the genus *Calvatia* with *Langermannia* taking a position of a subgenus [41].

Clade E presented a rather puzzling finding. The clade contained three sequences designated as “*B. dermoxantha*” (GenBank no. HQ235047 and no. HQ235050) and “*L. pusillum*” (GenBank no. AB067724), and received only Bayesian support (1.00 BPP). These three samples collected in the USA (NCP34 and SCP2) and Japan (Lp1, mycelia) during the studies of Miller et al. [67] and Terashima et al. [69] were related to the similar habitats—putting greens in golf courses. Intriguingly, the sequences clustered neither with *B. pusilla* in the *Bovista* clade C, nor with the sequences of *B. dermoxantha* (GenBank no. DQ112579) acquired by Larsson and Jeppson [41] and *B. dermoxantha* (OR594143) from the current research, which were nested within the major *Lycoperdon* clade (Figure 2). BLAST analysis showed that the sequences GenBank no. HQ235047, HQ235050, and AB067724 are fairly distant from other identified Lycoperdaceae sequences deposited in the GenBank database (less than 97% similarity), suggesting that they present a distinct species. Furthermore, the fact that the clade E took a position of a sister group to the *Lycoperdon* clade F reinforces this statement and even makes an argument for a separate subgenus/genus within Lycoperdaceae. However, this topology of the clade E was not statistically supported. Unfortunately, due to an obvious lack of morphological data regarding these three samples, we can only speculate on their taxonomical position.

Clade F in the current analysis represented the genus *Lycoperdon* s.l. The clade did not receive statistical support from either the ML or the Bayesian analyses. Thus, the problem of the genus monophyly remains under question. The clade splits into two subclades, F1 and F2, which also had no statistical support. The subclade F1 accommodated the type species of the genus, *L. perlatum*, with closely related *L. marginatum* and *L. norvegicum*. BLAST analysis showed that the sequence of *L. perlatum* (OR594101) obtained from the Israeli specimen is 99% similar to the sequence of *L. perlatum* GenBank no. DQ112630 [41], having one deletion, two substitutions, and one unidentified base pair difference. Sequences presenting Israeli and Swedish material of *L. perlatum* clustered in a clade with 100% BS and 1.00 BPP support. Additionally, within the subclade, a former representative of the genus *Vascellum*—*V. pratense*, two species both classified as belonging to the genus *Morganella*—*M. fuliginea* and *M. subincarnata*, one representative of the genus *Handkea*—*H. subcretacea*, and *L. caudatum* were nested. The close relationship of *L. perlatum*, *L. marginatum*, and *L. norvegicum* was supported by both ML (80% BS) and BI (0.99 BPP) methods. The subclade F2 united the major number of species within the *Lycoperdon* clade F. Apart from the species, which have been traditionally placed in the genus *Lycoperdon*, the subclade incorporated sequences assigned to the genera: *Calvatia*—*C. cretacea*, *C. turneri*; *Handkea*—*H. excipuliformis*, *H. fumosa*, *H. utriformis*; *Bovistella*—*B. radicata*; and the species *Holocotylon brandegeeanum*. The latter proved to be closely related to the members of the genus *Lycoperdon* by Bates et al. [43]. The sequences of *B. dermoxantha* (Swedish and German material), *L. rupicola*—the species recently described by Jeppson et al. [15], and *H. brandegeeanum* formed a well-supported clade in both ML and Bayesian analyses (72% BS/1.00 BPP). The subclade F2 corresponds with the subgenus *Utraria* (Quél.) Jeppson & E. Larss. proposed by Larsson and Jeppson [41], with the exception of *H. utriformis* and *B. radicata*.

In the light of the current research and previous molecular studies [14,15,21,41,43,44], Kreisel’s concept of the genus *Handkea* [77] appears to have a polyphyletic origin and presents an artificial taxon [78]. The main distinguishing characteristic of the genus, on the basis of which it was segregated from *Calvatia*, is slit-like pores in the capillitium. All the sequences under *Handkea* involved in the analysis, including *H. utriformis*, the type species of the genus, were nested within *Lycoperdon* clade F (Figure 2). The sequences of *B. radicata* (AJ237624 and OR594137), *C. utriformis* (OR594133), and *H. utriformis* (EU833659, DQ112607) clustered together in a well-supported clade (96% BS/1.00 BPP). We agree with Demoulin and Rebriev [78] and believe that the sequence GenBank no. AJ237624 [26] represents *C. utriformis* (= *H. utriformis*). A close phylogenetic relationship between *H. utriformis* and *B. radicata* was previously reported by Larsson and Jeppson [41]. The authors recovered them as members of *Lycoperdon* and proposed a separate subgenus *Bovistella* (Morgan) Jeppson & E. Larss. to accommodate *L. utriforme* and *L. radicatum*. The subgenus is characterized by medium-sized gasterocarps and the presence of a distinct pseudo-diaphragm. Analysis of Alfredo et al. supported this allocation [14]. Demoulin and Rebriev considered *B. radicata* and *C. utriformis* to be so closely related, both in terms of morphology and ribosomal locus, while at the same time being distinct enough from *Lycoperdon* s. str. that they should belong to the genus *Bovistella*, and introduced a new combination—*Bovistella utriformis* (Bull.: Pers.) Demoulin et Rebriev [78]. However, we believe that the exclusion of these two species from the genus *Lycoperdon* was rather premature, and this question requires more thorough molecular examination based on a wider taxonomical and geographical sampling, including *Bovistella japonica* Lloyd, *B. poeltii* Kreisel, *B. sinensis* Lloyd, etc.

*Lycoperdon atropurpureum* is probably the most common representative of the genus *Lycoperdon* in Israel. It is widespread in the woods throughout the northern region of the country, including the Carmel Mountain, the Western and the Eastern parts of Upper Galilee, the Golan Heights, and even the Hula Valley. Surprisingly, there were no specimens from the Lower Galilee region. Kreisel accepted a wide concept of *L. atropurpureum* and treated it and *L. decipiens* as synonyms [60]. Jeppson and Demoulin, however, demonstrated that there is a sufficient number of distinguishing macro- and micromorphological characteristics to consider *L. atropurpureum* and *L. decipiens* as separate species [79]. The authors included in their paper a comparative table, pointing out the differences between the two species. Pegler et al. also recognized *L. atropurpureum*, *L. decipiens*, and *L. molle* as distinct species, although very similar and often confusing [5]. Molecular phylogenetic reconstructions provided an additional support for their demarcation [21,41,44]. Depending on the method of tree construction, *L. atropurpureum* nested closer either to *L. decipiens* or *L. molle*. During the current study, fifteen sequences of *L. atropurpureum* were obtained (seven were included in the analysis): fourteen presented Israeli material and one originated from the French specimen (OR594124). BLAST analysis of all these sequences showed the closest match of 99% to the sequence of “*Lycoperdon* cf. *decipiens*” (GenBank no. DQ112586), which was obtained from the specimen of *L. atropurpureum* (M. Jeppson 3269) collected in Sweden [41]. The current analysis also recovers *L. atropurpureum*, *L. decipiens*, and *L. molle* as separate species, solidifying results of the earlier studies.

*Lycoperdon niveum* was described by Kreisel from the specimens collected in the Himalayas [80]. Later, the species was reported from Iceland, Norway, and Sweden (Gotland Island). Larsson and Jeppson noted that *L. niveum* belongs to the *L. molle* morphological species complex and its distribution is limited to arctic-alpine environments [41]. Alfredo provided molecularly verified findings of *L. niveum* from the Republic of Macedonia and Spain [81]. BLAST analysis showed that the sequence of Israeli specimen OR594103 is 99% similar (difference in two substitutions and one base marked as “W”) to the sequence of “*L.* cf. *niveum*” (GenBank no. DQ112571) acquired by Larsson and Jeppson [41] from *L. niveum* specimen (M. Jeppson 4068, Iceland). Both sequences of *L.* cf. *niveum* (OR594102 and OR594103) representing the material from Israel clustered with the sequence from Iceland in a clade with 74% BS and 0.99 BPP support (Figure 2). After analyzing both molecular and macro- and micromorphological data, the current Israeli material was identified as *L. niveum*. Thus, it must be noted that the range of *L. niveum* is broader than expected and its ecological and chorological data requires re-evaluation.

The specimen of *Lycoperdon* from Koncha-Zaspa, Ukraine (OR594091), collected and identified by Demoulin as “*L*. cf. *molle* Pers.”, turned out to be almost 100% similar (difference in only one base substitution) to GenBank sequence no. DQ112602 (“*L. lambinonii*” voucher MJ6371) representing recently described *L. subumbrinum* [15]. The species was originally reported from Sweden and Slovakia. Alfredo examined and sequenced *L. subumbrinum* specimens from France, the Republic of Macedonia, Spain, and the United Kingdom [81]. *Lycoperdon subumbrinum* is probably a widely distributed species, which had been misidentified in the past (as *L. molle*, *L. lambinonii*, etc.). The current study presents the first finding of the species from the territory of Ukraine. The species *L. subumbrinum*, *L. muscorum*, and *L. ericaeum* formed a clade with 98% BS and 1.00 BPP support. The same clade was recovered in the phylogenetic reconstructions of Larsson and Jeppson [41] and Jeppson et al. [15]. These species also clustered together in the analysis of Kim et al., along with newly described *Lycoperdon albiperidium* C.S. Kim [21].

In the course of the current study, six sequences from Israeli specimens (OR594118, OR594119, OR594120, OR594121, OR594122, and OR594123), presenting small puffballs, characterized by a compact subgleba, globose spores with short pedicels, and “*Lycoperdon*” type capillitium with abundant spherical to ellipsoid pores, were obtained. BLAST analysis revealed that the sequence OR594123 was the closest match to *L.* cf. *dermoxanthum* (GenBank no. FJ438478) from the molecular phylogenetic study of Bates et al. [43], demonstrating 99% identity, with a difference in one base insertion and three substitutions. The similarity with the sequence of *L. dermoxanthum* (= *B. dermoxantha*, GenBank no. DQ112579) acquired by Larsson and Jeppson [41] was only 98%—the sequence OR594123 had three insertions (one base each) and fourteen substitutions difference. The sequence from the specimen OR594119, which turned out to be the closest to *L.* cf. *dermoxanthum* (GenBank no. FJ438478), having one base deletion and one substitution difference, showed only 97% similarity with *L. dermoxanthum* (GenBank no. DQ112579). In the phylogenetic reconstructions presented by Bates et al., *L.* cf. *dermoxanthum* nested in a clade with “*L. niveum*” (*L. lividum* GenBank no. DQ112599) and *L. echinatum* [43]. The authors chose to treat the specimens from Arizona as *L.* cf. *dermoxanthum* until more information concerning this species is acquired. High similarities between the sequences obtained from the material collected in the USA and Israel resulted in a confusion, and Israeli samples were preliminarily identified as *L.* cf. *lividum*. Current molecular analysis recovered a clade uniting sequences of *L.* cf. *lividum* (Israel) and the sequence of *L.* cf. *dermoxanthum* (Arizona, USA) with the sequences of *L. lividum* (specimens from Belgium—GenBank no. OR594117 and Nepal—DQ112599). The clade was supported by both ML (80% BS) and BI (1.00 BPP) methods. The study of Bates et al. provided very similar descriptions and line drawings of *L. lividum* and *L.* cf. *dermoxanthum* [43]. Quality SEM micrographs of the material from Arizona also demonstrated the high similarity of these species in terms of basidiospore ultrastructure. Unfortunately, the authors did not include any sequences of *L. lividum* in their phylogenetic tree. More careful examination of Israeli material supported its earlier preliminary identification. Additionally, we believe that the specimens designated as *L.* cf. *demoxanthum* [43] most likely represent *L. lividum*.

In summary, phylogenetic relations within the family Lycoperdaceae remain only partly resolved. However, the data accumulated during the current and previous molecular studies [2,14,15,18,21,26,40,41,43,44,45,46,47,78] allow us to draw some preliminary conclusions. Lycoperdaceae appears to be a monophyletic group comprised of puffball-like species, including the type genus *Lycoperdon* and its allies. Within Lycoperdaceae, clades corresponding to the genera *Apioperdon*, *Bryoperdon*, *Bovista*, *Calvatia*, *Disciseda*, and *Lycoperdon* can be distinguished. Yet, their relative position is still debatable. *Apioperdon* seems to present a distinct monotypic genus within the family, although its exact placement is also questionable. The main uncertainty lies within the genus Lycoperdon, which most likely represents a polyphyletic entity. The current analysis supported the inclusion of the following species commonly assigned to the related genera into *Lycoperdon* s.l.: *Handkea fumosa*, *Holocotylon brandegeeanum*, *Lycoperdon cretaceum*, *L. dermoxanthum*, *L. excipuliforme*, *L. fuligineum*, *L. pratense*, *L. radicatum*, *L. subincarnatum*, *L. subcretaceum*, *L. turneri*, and *L. utriforme*. This shows that *Lycoperdon* is a taxon with higher morphological variability than was formerly accepted, and it can be left intact in a form of a single unit only if a broader genus concept is proposed. Another option would be to establish the monophyletic *Lycoperdon* by reducing its diversity to the generic type and limited number of related species [41]. The polyphyletic nature of the genus requires more in-depth investigations, because we are still lacking a solid statistically supported picture regarding the species composition and relationships within *Lycoperdon*. Similar problems arise when dealing with the family Lycoperdaceae. If it is incorporated into the family Agaricaceae, the rank of Lycoperdaceae could potentially be lowered to the tribe level, as was proposed by Larsson and Jeppson [41]. *Mycenastrum corium* appears to be a sister taxon to Lycoperdaceae and could be treated as a monotypic tribe within the family Agaricaceae.

Both individual ITS and combined ITS + partial nuc-LSU datasets proved to be insufficient to generate phylograms that fully resolve phylogenetic relations within Lycoperdaceae, while providing significant statistical support. It is noteworthy to mention that ITS and nuc-LSU sequences of different Lycoperdaceae representatives demonstrated very high similarities. The difference between related species in the ITS gene is usually less than 1%. This is even more relevant for the nuc-LSU region, where such dissimilarity could come down to several base pairs. It is now clear that future investigations of gasteroid lineages within the Agaricales, and the “puffball” lineage in particular, will depend on the multigene phylogenies, which will give greater resolution and provide higher levels of statistical support. RNA polymerase II gene subunits RPB1 and RPB2, translation elongation factor 1 alpha (TEF-1α), intergenic spacer (IGS), and mitochondrial rDNA genes are the most likely candidates for additional molecular phylogenetic studies. Furthermore, RAPD (Random Amplified Polymorphic DNA) and microsatellite analyses can be used for the purpose of resolving phylogenetic relations within Lycoperdaceae. The studies of Jeppson et al. [82,83] dealing with phylogenetic relationships within the gasteroid family Geastraceae and the genus *Tulostoma* Pers., both based on combined full ITS, partial LSU, and partial TEF-1α datasets, are examples of modern tendencies in fungal molecular systematics. The contribution of morphological and molecular features in creating a self-consistent taxonomic picture of both individual genera, and Lycoperdaceae in general, remains debatable and requires further studies.

### 3.2. Taxonomy

**Figure 3:**
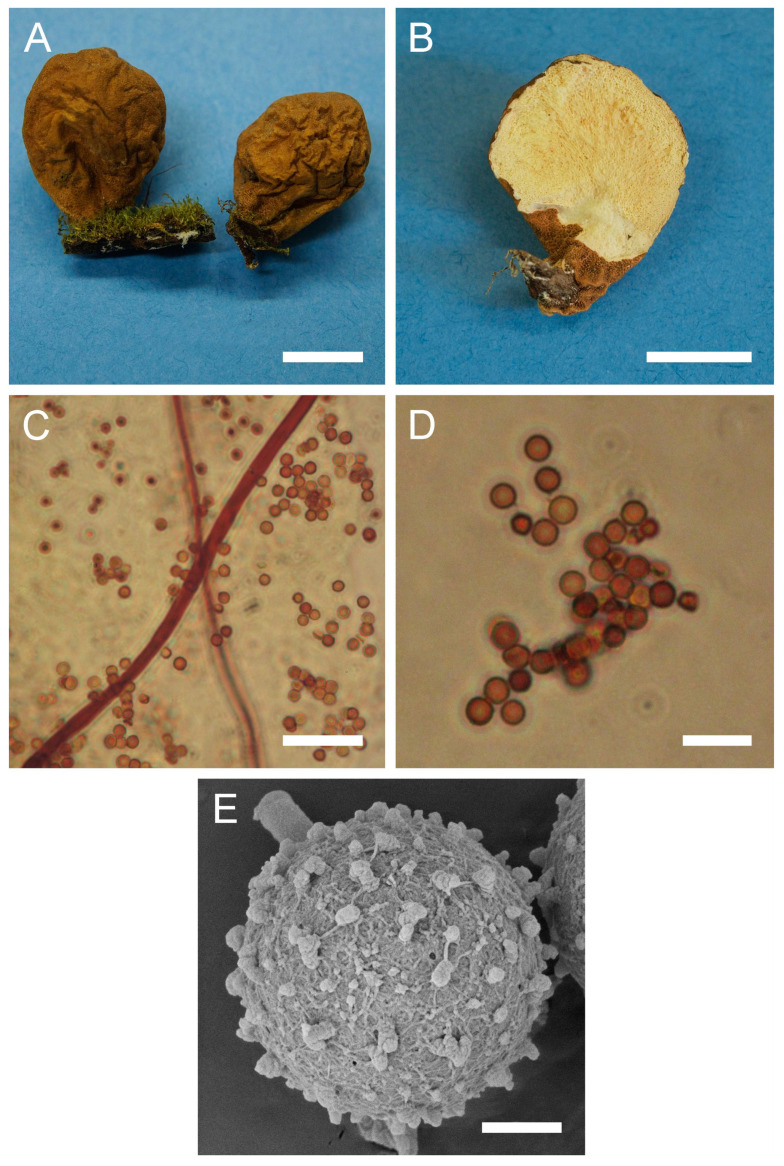
Macro- and micromorphology of *A. pyriforme* (HAI-G-11, HAI-G-159). (**A**) Gasterocarps (scale bar = 1 cm). (**B**) Cross-section of the gasterocarp (scale bar = 1 cm). (**C**) Basidiospores and capillitium hyphae under light microscopy (Melzer’s reagent, scale bar = 20 µm). (**D**) Basidiospores under light microscopy (Melzer’s reagent, scale bar = 10 µm). (**E**) Microstructure of the basidiospore under SEM (scale bar = 1 µm).

**Figure A1:**
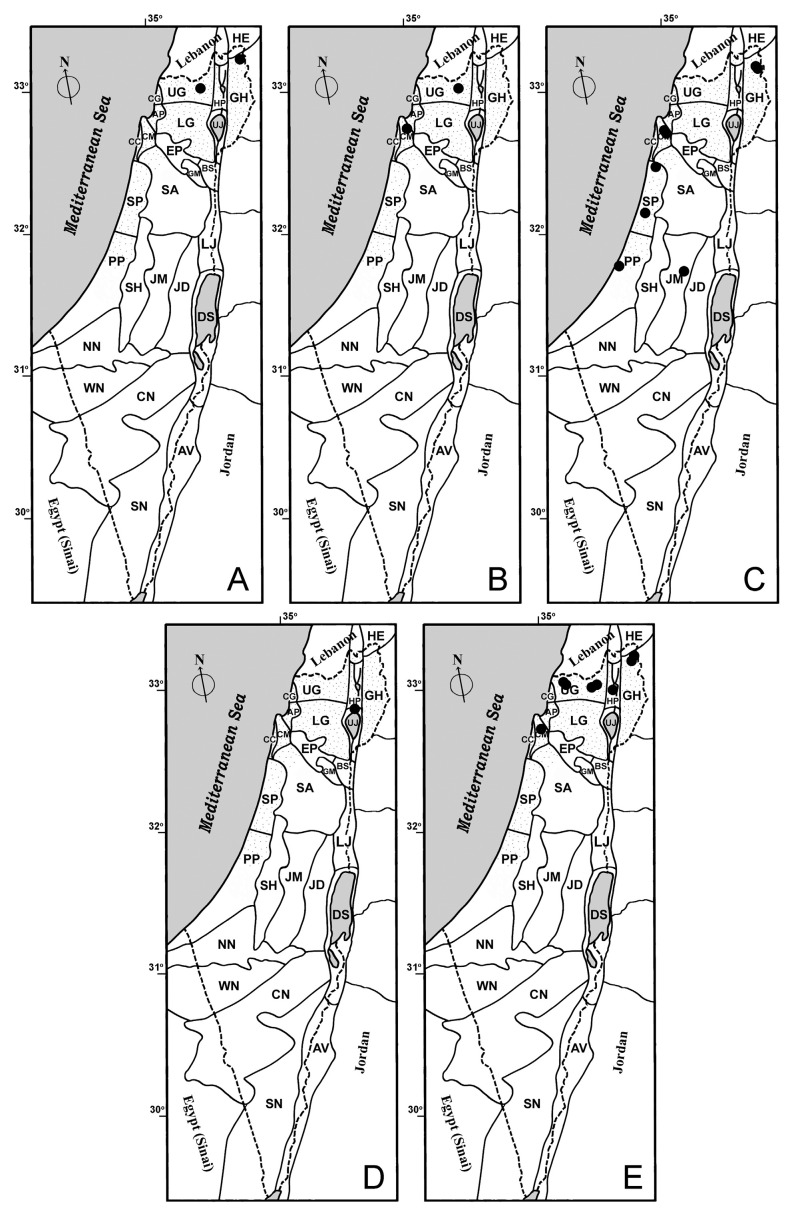
Distribution of Lycoperdaceae species in Israel. (**A**) *Apioperdon pyriforme*; (**B**) *Bovista aestivalis*; (**C**) *B. plumbea*; (**D**) *Calvatia candida*; (**E**) *Lycoperdon atropurpureum*.

Genus: ***Apioperdon*** (Kreisel & D. Krüger) Vizzini, Phytotaxa 299: 81 (2017)
Type species: *Apioperdon pyriforme* (Schaeff.) Vizzini, Phytotaxa 299: 81 (2017)
***Apioperdon pyriforme*** (Schaeff.) Vizzini, Phytotaxa 299: 81 (2017). Figure 3 and Figure A1A.

Basionym: *Lycoperdon pyriforme* Schaeff., Fung. Bavar. Palat. 4: 128 (1774)

Synonyms: *Utraria pyriformis* (Schaeff.) Quél., Mémoires de la Société d’Émulation de Montbéliard, 5: 369 (1873); *Morganella pyriformis* (Schaeff.: Pers.) Kreisel & D. Krüger, Mycotaxon, 86: 175 (2003); *Lycoperdon pyriforme* Willd., Florae Berolinensis Prodromus: 411 (1787); *Lycoperdon pyriforme* var. *tessellatum* Pers., Syn. Meth. Fung.: 149 (1801); *Lycoperdon pyriforme* Vent.: index, t. 32 (1812); *Scleroderma bresadolae* Schulzer, Hedwigia, 23: 163 (1884)

Description: Gasterocarps growing on wood in clusters, pyriform, 2.5–3 cm in height and 1.5–2 cm in width, with white rhizoids. Exoperidium 12 (Fulvous) to 17 (Snuff brown) with minute warts, somewhat granulose, which is more distinct down towards the base. Endoperidium papery, light brown to reddish brown. Pseudocolumella present. Gleba first white 4D-5E, then olive to grey-brown. Subgleba whitish 2B. Capillitium from 2–3 μm (wall 0.4–0.5 μm), yellow, to 4–5.5 (up to 6 μm), reddish brown in Melzer’s reagent (wall 0.6–0.7, rarely up to 1 μm). Pores not observed. Spores globose to subglobose (3) 3–3.6 (4) μm in diam. (*n* = 30), under light microscopy appear smooth, light reddish brown in Melzer’s reagent, some with a small pedicel attached (usually less than 1 μm long, sometimes up to 2–3 μm). Sphaerocysts 35–45 × 25–30 μm, subglobose to ovoid-pyriform.

Under SEM spores appear globose to subglobose, with a prominent ornamentation of low apprised warts irregular in shape and with rounded and flattened tips. Some warts are merged together or rarely connected by low, thin anastomoses. The spore surface between warts looks rough and rugged, and has numerous small warts and ornamentation, comprised of somewhat short strands. Apiculus usually 0.5–0.7 μm long is observed.

Habitat: Lignicolous species found in broad-leaved, coniferous and mixed conifer forests, parks and gardens on decaying wood (logs, stumps, etc.), mostly hardwood, but also softwood. Sub-cosmopolitan species present in all continents, except Africa and Antarctica.

General distribution: Europe: Andorra, Austria, Denmark, Germany, Finland, Italy, Ireland, Luxembourg, Norway, Poland, Romania, Russian Federation (Central European part), Slovenia, Spain, Sweden, Switzerland, United Kingdom. Middle East: Israel. Asia: Japan. North America: Canada, Mexico, USA. Central America: Costa Rica. South America: Argentina. Arctic Territories: Iceland. Australasia: Australia, New Zealand.

Material examined: Israel. UG. Mt. Meron National Park. On wood. 7 January 2004. *Leg*. Y. Ur, *det*. M. Krakhmalnyi (HAI-G-11). GH. Forest Odem. On wood. 8 December 2012. *Leg*. Z. Shafranov, A. Biketova, *det*. M. Krakhmalnyi (HAI-G-159).

Notes: For a long time, the taxonomic position of the species remained debatable. In 2003, Krüger and Kreisel published an article in which they transferred *L. pyriforme* to the lignicolous genus *Morganella*, creating a new combination—*Morganella pyriformis* (Schaeff.: Pers.) Kreisel & D. Krüger [45]. The authors also proposed a new subgenus *Apioperdon* Kreisel & D. Krüger, to accommodate *M. pyriformis*. The subgenus took a somewhat intermediate position between *Lycoperdon* and *Morganella* subgen. *Morganella* in terms of its morphological features and habitat. Subsequent studies pertaining to molecular phylogenetics of the family Lycoperdaceae were not congruent with the results of Krüger and Kreisel [45]. In the reconstructions of Larsson and Jeppson, *L. pyriforme* occupies a basal position to the *Lycoperdon* clade. Additionally, the species did not cluster with sequences of *M. fuliginea* (Berk. & M.A. Curtis) Kreisel & Dring and *M. subincarnata* (Peck) Kreisel & Dring, which represented the genus *Morganella* in the analysis. Bates et al. [43], also demonstrated that *L. pyriforme* is not phylogenetically related to representatives of *Morganella*, and the authors chose not to follow Krüger and Kreisel [45] in placing the species in the latter genus, although they totally agreed with the fact that the lignicolous habit of *L. pyriforme* could be a strong case for putting it into *Morganella*. The current phylogenetic analysis supports the earlier results of Vizzini and Ercole [2].

The SEM micrographs supplied by Krüger and Kreisel show spores with low isolated warts with rounded apices, and low conical processes [45]. SEM photos of Bates et al. demonstrated similar morphology, with spores having ornamentation of very low warts [43]. Spores of current samples have notably denser ornamentation of more pronounced warts, in addition to the whole spore surface being rough and rugged.

**Figure 4:**
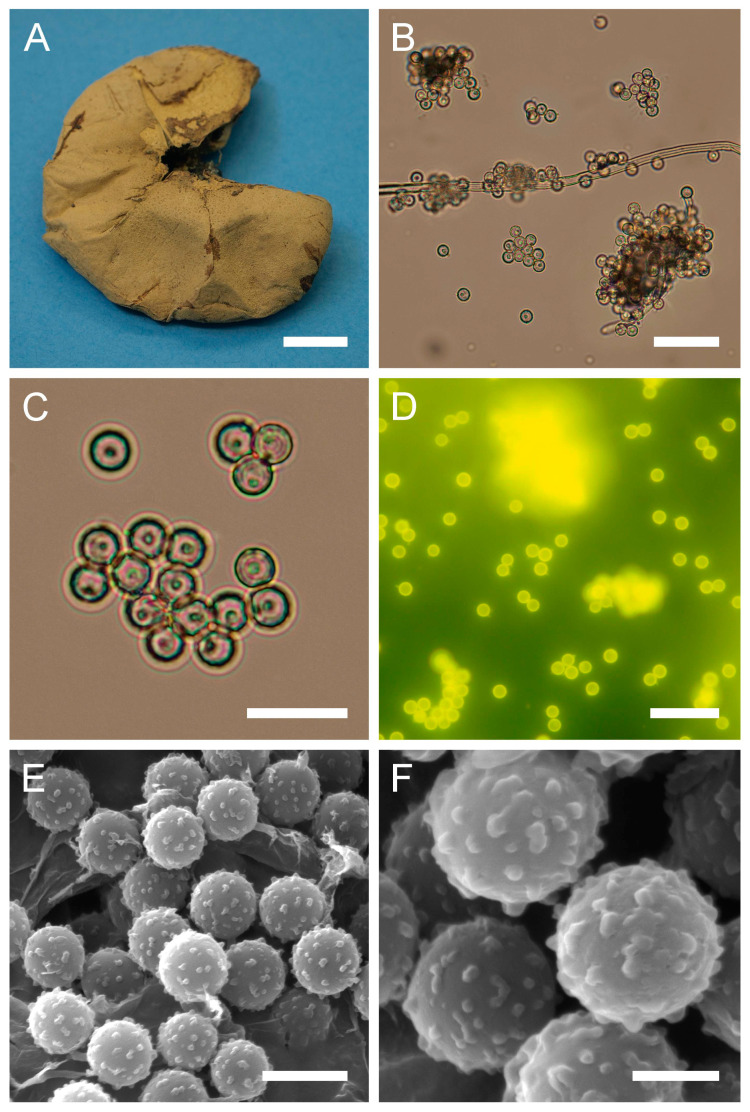
Macro- and micromorphological characteristics of *B. aestivalis* (HAI-G-73). (**A**) Dried gasterocarp (scale bar = 1 cm). (**B**) Basidiospores and capillitium hypha under light microscopy (scale bar = 20 µm). (**C**) Basidiospores under light microscopy (scale bar = 10 µm). (**D**) Basidiospores under light microscopy (epifluorescence mode, scale bar = 20 µm). (**E**) Basidiospores under SEM (scale bar = 5 µm). (**F**) Microstructure of basidiospores under SEM (scale bar = 2 µm).

Genus: ***Bovista*** Pers., Neu. Mag. Bot. 1: 86 (1794)
Type species: Bovista *plumbea* Pers.: Pers., Obs. Mycol. 1: 5 (1796)
***Bovista aestivalis*** (Bonord.) Demoulin, Beih., Sydowia 8: 143 (1979). Figure 4 and Figure A1B.

Basionym: *Lycoperdon aestivale* Bonord., Handb. Allgem. mykol. (Stuttgart): 251 (1851)

Synonyms: *Lycoperdon furfuraceum* Schaeff., Fung. Bavar. Palat. Nasc. 4: 131 (1774), non *Bovista furfuracea* Pers.: Pers. 1801; *Lycoperdon cepiforme* Bull., Annales de l’Institut agronomique de Moscou: 156 (1791) var. *cepiforme*; *Lycoperdon polymorphum* Vittad., in Vittadini C. Monogr. Lycoperd., Mem. Accad. Torino, vol. 5: 39 (1842), nom. illegit, non *Lycoperdon polymorphum* Scop. 1772; *Lycoperdon coloratum* Peck, Rep. State Bot. of New York State Mus.: 29: 46 (1878); *Lycoperdon ericetorum* var. *cepiforme* (Bull.) Bowerman [as ‘*cepaeforme*’], Can. J. Bot. 39: 364 (1961); *Bovista colorata* (Peck) Kreisel, Feddes Repert. 69: 201 (1964); *Bovista polymorpha* (Vittad.) Kreisel, Reprium nov. Spec. Regni veg. 69: 201 (1964); *Bovista pusilliformis* (Kreisel) Kreisel, Feddes Repert. 69: 202 (1964); *Bovista aestivalis* (Bonord.) Demoulin, Beih., Sydowia 8: 143 (1979) var. *aestivalis*

Description: Gasterocarps (3) 4–5 cm in diam., subglobose or pyriform, with 0.5–1 cm basal turf of mycelium intermixed with soil particles. Exoperidium almost smooth, covered with fine spines, especially towards the base of the gasterocarp. Color 5E (6F) to 52 (Buff), some mature specimens 17 (Snuff brown) to 27 (Hazel), with little purple-reddish tint. Endoperidium papery, yellow to 27 (Hazel). Gleba 27 (Hazel) to 62 (Olivaceous). Spore print olivaceous-yellow. Under light microscopy, basidiospores (3) 3.6–4.4 (4.5) µm in diam. (*n* = 38), globose, finely ornamented, hyaline to light green-yellow. Most of the spores have short pedicel (usually less than 1–1.5 µm). Capillitium hyphae of intermediate type, from 2–3.5 µm in width, hyaline to light green-yellow (wall ≈ 0.5 μm), to darker in color 4–5 (up to 6) µm in width (wall ≈ 0.7, up to 1 µm). Old thick hyphae with incrustations.

Under SEM, spores almost perfectly globose, covered with low, irregularly shaped warts, usually with rounded apices and ridges. Warts almost evenly dispersed throughout the spore surface, sometimes merged together or rarely connected by low thin anastomoses. Apiculus 0.5–1.7 µm in length and 0.5 µm in diam., with a distinct terminal pore, is observed. Area surrounding apiculus usually less crowded with warts or completely free of any ornamentation. Additionally, in the current research, spores and capillitium of some specimens were studied in epifluorescence mode. In all the samples, it was observed that spores shine intensively under ultraviolet light; as for the hyphae, they appear almost dark.

Habitat: The species prefers warm and dry habitats, can be found in open areas on calcareous or, less frequently, sandy soils and sand fields, sand dunes, sand steppes, and dry meadows vegetation in the communities of *Festucetalia vaginatae* (records from Hungary), or grows amid leafy debris under *Abies* spp., *Juniperus* spp., *Pinus* spp., etc.

Material examined: Israel. UG. Mt. Meron National Park. On the ground, under *Pinus* sp. 19 March 1997. *Leg*.—, *det*. M. Krakhmalnyi (HAI-G-73). CM. Mt. Carmel National Park. On the ground. 10 February 2012. *Leg*. O. Godorova, *det*. M. Krakhmalnyi (HAI-G-103).

General distribution: Europe: Estonia, France (South and Central), Germany, Hungary, Netherlands, Scandinavia (includes three kingdoms of Denmark, Norway, Sweden, Finland), Spain, United Kingdom. Arctic Territories: Iceland. Middle East: Israel, Turkey. Asia: China, Japan, Mongolia. (Jeppson (2001) noted that records from North America, Africa, Australia, and New Zealand should be treated carefully and may refer to closely related taxa).

Notes: *Bovista aestivalis* is a very polymorphous species with a wide range of distribution and habitats, which consequently resulted in many issues related to its identification, synonyms and demarcation from morphologically similar species. A complicated situation with synonyms is still misleading in terms of creating a global map of its distribution. Demoulin synonymized *B. pusilliformis* (Kreisel) Kreisel and *B. polymorpha*, creating a new combination—*B. aestivalis* (Bonord.) Demoulin [84]. He found that observed morphological differences between the named taxa mostly depend on the environmental factors, and on the portion of gleba from which the sample was taken for the examination of the capillitium structure. Some findings of *B. aestivalis* were recorded as *Lycoperdon furfuraceum* Hollós [85]. Jeppson, in his survey of *B. aestivalis* occurrence in northern Europe, presented a very wide discussion on variations in macro- and micro-characteristics of the studied material [86]. He demonstrated that gasterocarps of *B. aestivalis* could be small and subglobose, possessing a very small, reduced sterile base (subgleba), or, to the contrary, some samples could have well developed subgleba, forming a pseudostipe, which comprises almost half a length of the gasterocarp. The sample HAI-G-73 has a subglobose shape of the gasterocarp with a small-to-reduced subgleba, and the sample HAI-G-103 is pyriform with a developed pseudostipe. Additionally, the color of the gasterocarps widely varies.

Moyersoen and Demoulin studied a large number of *B. aestivalis* specimens from Corsica under SEM, and presented photomicrographs reveal some polymorphism of spores’ ultrastructure [63]. In their specimens, spores tend to be slightly ovoid in shape (in the current material, spores are globose), a larger number of spores have numerous meshes of low, thin ornamentation between warts. Some of the specimens from Corsica have spores with denser ornamentation of warts merging together and forming irregularly shaped conglomerates; also, some spores tend to have a pattern of small warts surrounding larger cylindrical in shape warts with rounded or flattened tips. Current SEM photos are in agreement with SEM micrographs and descriptions of Rimóczi et al. [65].

***Bovista nigrescens*** Pers., Neues Mag. Bot. 1: 86 (1794)

Synonyms: *Bovista nigrescens* Pers., Neues Mag. Bot. 1: 86 (1794) subsp. *nigrescens*; *Bovista nigrescens* Pers., Neues Mag. Bot. 1: 86 (1794) var. *nigrescens*; *Sackea nigrescens* (Pers.) Rostk., Deutschl. Fl., 3 Abt. (Pilze Deutschl.) 5(18): 33 (1839); *Lycoperdon nigrescens* (Pers.) Vittad., in Vittadini C. Monogr. Lycoperd., Mem. Accad. Torino, vol. 2: 176 (1843); *Globaria nigrescens* (Pers.) Quél., Mém. Soc. Émul. Montbéliard, Sér. 2, 5: 372 (1873); *Bovista nigrescens* subsp. *tenella* Sacc., Michelia 2(no. 8): 565 (1882); *Bovista nigrescens* var. *tenella* (Sacc.) Sacc., Syll. fung. (Abellini) 7: 99 (1888); *Bovista montana* Morgan, J. Cincinnati Soc. Nat. Hist. 14: 145 (1892); *Bovista nigrescens* var. *asperispora* Moesz, Bot. Közl. 35(1–2): 66 (1938); *Bovista nigrescens* var. *montana* (Morgan) F. Šmarda, Fl. ČSR, B-1, Gasteromycetes: 364 (1958)

Habitat: Terricolous in open grassy areas—meadows, pastures, rarely in cultivated fields and on old sewage beds, also in rich deciduous woodland, dunes and mountains above timberline; in subarctic to subtropical climates.

General distribution: Europe: Andorra, Austria, Bulgaria, Czech Republic, Denmark, Finland, France, Germany, Greece, Ireland, Italy, Lithuania, Netherlands, Norway, Poland, Romania, Russian Federation (European part), Slovakia, Spain, Sweden, Switzerland, Ukraine, United Kingdom. Mediterranean Islands: Corse (France). Arctic Territories: Greenland, Iceland. Middle East: Armenia, Azerbaijan, Georgia, Israel. Asia: China. South America: Argentina, Ecuador.

Previous records from Israel: Sharon Plain. Pardes Hanna. 1938 (Kew) [49].

Notes: The species can sometimes be confused with *B. plumbea*, although they are distinguished by a number of macro- and micromorphological characteristics. Endoperidium of *B. nigrescens* is dark brown, while in *B. plumbea* it is lead-grey. Gasterocarps of *B. nigrescens* are, on average, larger than specimens of *B. plumbea*. Microscopic differences include more ovoid shape of the spores and notably longer sterigmal remnants in *B. plumbea*.

**Figure 5:**
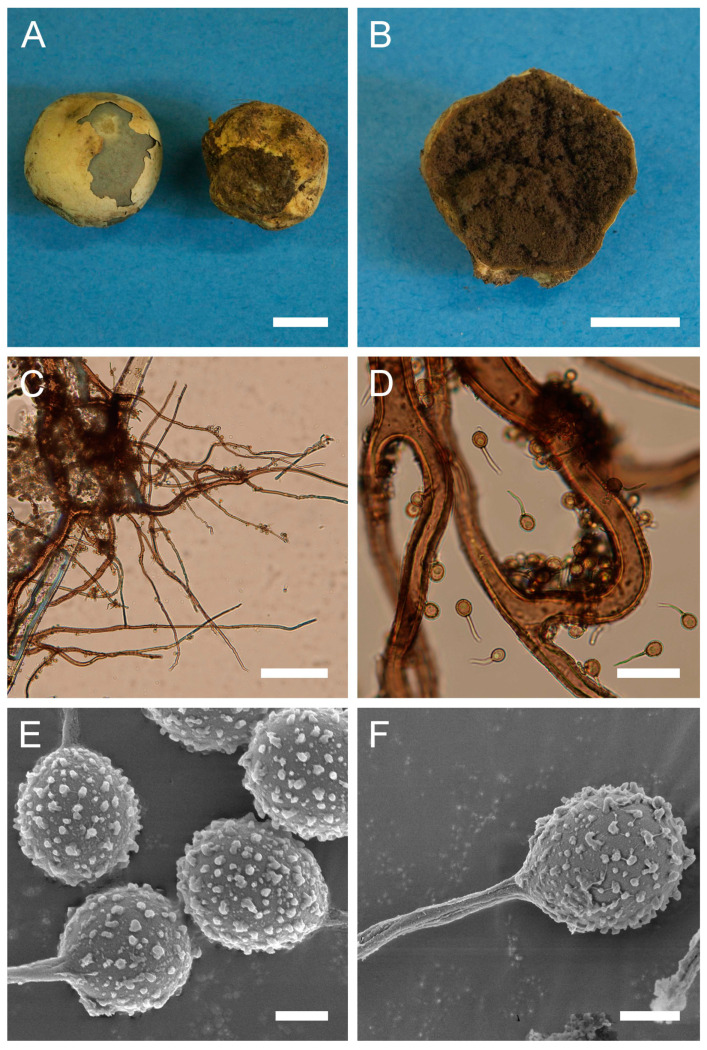
Macro- and micromorphology of *B. plumbea* (HAI-G-24, HAI-G-218). (**A**) Gasterocarps (scale bar = 1 cm). (**B**) Cross-section of the gasterocarp (scale bar = 1 cm). (**C**) Capillitium (“*Bovista*” type) under light microscopy (scale bar = 100 µm). (**D**) Basidiospores and capillitium hyphae under light microscopy (scale bar = 20 µm). (**E**) Microstructure of basidiospores under SEM (scale bar = 2 µm). (**F**) Single basidiospore with a long pedicel (scale bar = 2 µm).

***Bovista plumbea*** Pers., Ann. Bot. (Usteri) 15: 4 (1795). Figure 5 and Figure A1C.

Synonyms: *Bovista plumbea* Pers., Ann. Bot. (Usteri) 15: 4 (1795) var. *plumbea*; *Bovista plumbea* Pers., Ann. Bot. (Usteri) 15: 4 (1795) f. *plumbea*; *Lycoperdon bovista* Sowerby, Col. Figure Engl. Fung. Mushr. (London) 3: pl. 331 (1803); *Bovista suberosa* Fr., Syst. mycol. (Lundae) 3(1): 26 (1829); *Lycoperdon plumbeum* Vittad., in Vittadini C. Monogr. Lycoperd., Mem. Accad. Torino, vol. 5: 174 (1842); *Endonevrum suberosum* (Fr.) Czern., Bull. Soc. Imp. Nat. Moscou 18(2, III): 151 (1845); *Globaria plumbea* (Pers.) Quél., Mém. Soc. Émul. Montbéliard, Sér. 2 5: 371 (1873); *Globaria plumbea* (Pers.) Quél., Mém. Soc. Émul. Montbéliard, Sér. 2 5: 371 (1873) var. *plumbea*; *Globaria plumbea* var. *suberosa* (Fr.) Quél., Mém. Soc. Émul. Montbéliard, Sér. 2 5: 371 (1873); *Bovista ovalispora* Cooke & Massee, Grevillea 16(no. 78): 46 (1887); *Bovista brevicauda* Velen., České Houby 4–5: 832 (1922); *Bovista plumbea* var. *flavescens* Hruby, Hedwigia 70: 350 (1930); *Bovista plumbea* var. *brevicauda* (Velen.) F. Šmarda, Fl. ČSR, B-1, Gasteromycetes: 367 (1951); *Bovista plumbea* f. *brevicauda* (Velen.) F. Šmarda, Fl. ČSR, B-1, Gasteromycetes 12: 239 (1958); *Bovista plumbea* var. *ovalispora* (Cooke & Massee) F. Šmarda, Fl. ČSR, B-1, Gasteromycetes 12: 239 (1958)

Description: Gasterocarps usually in small groups, subglobose, compressed either from the sides or from top to bottom, 2–3.5 (up to 5) cm in height and 2–3 (up to 5.5) cm in width, with 0.5–0.7 cm rhizomorph binding soil particles. Exoperidium almost smooth, 3C to 5E in color, sometimes with a reddish tint. After maturation, exoperidium becomes very thin and wears off like layers of old paint, revealing a mat 34 (Smoke grey) endoperidium. Mature gleba 16 (Cigar brown), 17 (Snuff brown) to 26 (Sepia). Spore print brown to sepia. Capillitium of “*Bovista*” type, light brown, with main branches up to 15–20 µm in diam., thick-walled, non-poroid, non-septate. Basidiospores 4.5–6 × 4–5.5 µm, subglobose to ovoid, yellow to light brown, finely asperulate under light microscopy. Under SEM, the spore surface is covered with dense ornamentation of low irregular warts, occasionally connected by thin anastomoses. Pedicels 7–15 µm long are observed.

Habitat: Terricolous among short grass in open areas—meadows, pastures, golf links, lawns, downland and roadside verges, often in coastal turf and dunes, rarely in open woodland; in subarctic to subtropical climates.

General distribution: Europe: Andorra, Austria, Belgium, Denmark, France, Germany, Greece, Hungary, Ireland, Italy, Luxembourg, Norway, Poland, Slovenia, Romania, Russian Federation (European part), Spain, Sweden, Switzerland, United Kingdom. Mediterranean Islands: Balearic Islands (Spain). North America: Canada, USA. Caribbean region: Dominican Republic. South America: Argentina. Arctic Territories: Iceland. Asia: China, Mongolia, Pakistan. Australasia: New Zealand.

Material examined: Israel. CM. Mt. Carmel National Park, “Little Switzerland”. On the ground. 21 February 2010. *Leg*. P. Tovbin, *det*. M. Krakhmalnyi (HAI-G-8). GH. Masaade forest, near Buq’ata. Under *Quercus* sp. 4 January 1995. *Leg*. S.P. Wasser and E. Nevo, *det*. M. Krakhmalnyi (HAI-G-24). CM. Horshat HaArbaim. On the ground. 3 February 2003. *Leg*. S.P. Wasser, *det*. M. Krakhmalnyi (HAI-G-25). GH. Masaade forest. On the ground. 21 January 1995. *Leg*. S.P. Wasser, *det*. M. Krakhmalnyi (HAI-G-68). CM. Mt. Carmel National Park. On the ground. 15 February 2012. *Leg*. O. Godorova, *det*. M. Krakhmalnyi (HAI-G-172). GH. Masaade forest. On the ground. 7 April 2007. *Leg*. Z. Shafranov, *det*. M. Krakhmalnyi (HAI-G-181). PP. Near Ashdod. On the ground. 8 November 2012. *Leg*. A. Gibkhin, *det*. M. Krakhmalnyi (HAI-G-218).

Notes: Type species of the genus, widespread and very common. Characterized by a lead grey endoperidium, lack of subgleba, and subglobose to ovoid spores with long sterigmal remnants. Phylogenetic study of Larsson and Jeppson demonstrated that sequenced representatives of the genus *Bovista* form a clade that received a moderate support in Bayesian analysis [41]. However, this clade splits into two well-supported subclades roughly corresponding to the subgenera *Bovista* and *Globaria*. *Bovista plumbea* nested within the subgenus *Bovista* clade, which includes species characterized by “*Bovista*” type dichotomously branched capillitium and pedicellate spores. *Bovista limosa* Rost. turned out to be the closest relative of *B. plumbea* in the *Bovista* clade.

***
Bovista pusilla*** (Batsch) Pers., Syn. meth. fung. (Göttingen) 1: 138 (1801)

Basionym: *Lycoperdon pusillum* Batsch, Elench. fung., cont. sec. (Halle): 123, Table 41:228 (1789)

Synonyms: *Globaria pusilla* (Pers.) Quél., Mém. Soc. Émul. Montbéliard, Sér. 2 5: 371 (1873); *Pseudolycoperdon pusillum* (Pers.) Velen., Novit. Mycol. Nov., (Op. Bot. Ćech.): 93 (1947); *Lycoperdon polymorphum* var. *pusillum* (Pers.) F. Šmarda, Fl. ČSR, B-1, Gasteromycetes: 238 (1958)

Habitat: Terricolous in open areas—fields, meadows, road verges, in dunes on poor sand and limestone soils, in subarctic to tropical climates.

General distribution: Europe: Austria, Denmark, Germany, Norway, Romania, Russian Federation (European part), Spain, Sweden. Asia: China? Indian Ocean: Mauritius. North America: Canada, USA. (Distribution should be treated carefully, since some findings could be referred to related taxa. For instance, arctic samples of “*Bovista pusilla*” represent *B. limosa* Rostr.).

Previous records from Israel: Sharon Plain, Pardes-Hanna, in grassy places. 15 December 1957 (as *Lycoperdon pusillum* [49]).

Notes: The concept of *B. pusilla* united small bovists with absent or little developed subgleba, “*Lycoperdon*” type capillitium and small spores with a short apiculus. Dring and Rayss reported the presence of *Lycoperdon pusillum* Batch in Israel [49]. The situation around this finding still remains confusing. The authors gave a short description of a specimen from Pardes-Hanna (Sharon Plain) without providing any photos or illustrations of its macro- and micromorphology. The description of Dring and Rayss [49] is close to the one of *B. dermoxantha* presented in the work of Pegler et al. [5]. The latter pointed out that *B. pusilla* should be considered a *nomen ambiguum*.

*Lycoperdon dermoxanthum* was described by Hollós as *L. hungaricum* [87]. Kreisel [88] in his wide recognition of *B. pusilla* incorporated *B. dermoxantha* and *B. furfuracea*, but twenty years later, Moyersoen and Demoulin [63] chose to divide *B. pusilla* into *B. furfuracea* and *B. dermoxantha*, thus re-establishing them as separate species. The authors based this recognition on macro- and micromorphological characteristics, including gasterocarp morphology (the presence of whitish subgleba in *B. dermoxantha*), and spore and capillitium structure. Additionally, they studied spore micromorphology of a large number of specimens under SEM.

Modern methods of molecular biology helped to resolve this situation. In 2008, Larsson and Jeppson published their study on the phylogenetic relations within the family Lycoperdaceae [41]. The sequence of *B. dermoxantha* (GenBank no. DQ112579), one sequence of *B. pusilla* (GenBank no. AJ237631) originated from the study of Krüger et al. [26], and the sequence of *B. furfuracea* (GenBank no. DQ112622) were included in the analysis. The study clearly demonstrated that sequences of *B. dermoxantha*, *B. furfuracea* and *B. aestivalis* present distinct species. The last two sequences nested in a clade corresponding to the subgenus *Globaria* within the genus *Bovista*, while *B. dermoxantha* appeared in the *Lycoperdon* clade separately to all *Bovista* species. High similarity between the sequences of *B. pusilla* and *B. furfuracea* were discussed and the authors suggested that Krüger et al. [26], in their identification, followed a concept of *B. pusilla* used by Kreisel. The sequence of *B. furfuracea* (GenBank no. DQ112622) showed a huge separation from *B. dermoxantha* supporting morphological demarcation of the species by Moyersoen and Demoulin [63]. Later, Larsson et al. demonstrated that *B. pusilla* and *B. limosa* represent two distinct, yet closely related species, which nested within the subgenus *Bovista* [47].

Due to the objective lack of data, it is very difficult to say now what species was really observed by Dring and Rayss [49]: it could have been *B. furfuracea*, *L. dermoxanthum*, *B. aestivalis* or, maybe, something similar. Thus, the current study chose to treat the finding as *B. pusilla* until more data will be obtained.

**Figure 6:**
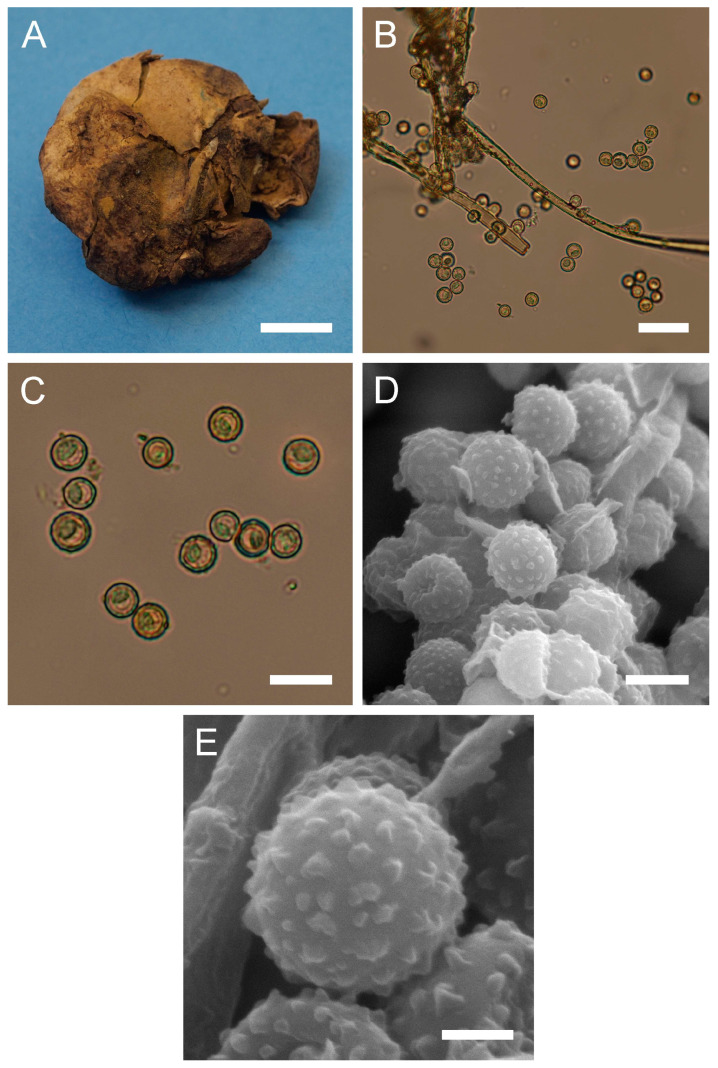
Macro- and micromorphology of *C. candida* (HAI-G-78). (**A**) Dried gasterocarp (scale bar = 1 cm). (**B**) Basidiospores and capillitium hyphae under light microscopy (scale bar = 20 µm). (**C**) Basidiospores under light microscopy (scale bar = 10 µm). (**D**) Basidiospores under SEM (scale bar = 5 µm). (**E**) Microstructure of the basidiospore under SEM (scale bar = 1 µm).

Genus: ***Calvatia*** Fr., Summa Veg. Scand., Section Post. (Stockholm): 442 (1849)
Type species: *Calvatia craniiformis* (Schwein.) Fr., Summa Veg. Scand.: 442 (1849)
***Calvatia candida*** (Rostk.) Hollós, Term. Füz. 25: 112 (1902). Figure 6 and Figure A1D.

Basionym: *Langermannia candida* Rostk., Deutschl. Fl., 3 Abt. (Pilze Deutschl.) 5(18): 25 (1839)

Synonyms: *Lycoperdon candidum* (Rostk. 1839) Saccardo, Syll. Fung. VII: 483 (1888); *Bovista olivacea* Cooke & Massee, Grevillea 16 (79): 77 (1888); *Lycoperdon hungaricum* Velenovský, České houby: 820 (1922)

Description: Gasterocarps subglobose, (2) 4–4.5 cm in width, (2) 2.5 (3) cm in height. Color 52 (Buff) at the base, tending to become 17 (Snuff brown) to 18 (Umber) towards the top with a reddish tint. Mature gleba olivaceous-yellow. Under light microscopy, spores globose to slightly subglobose, (4) 4.9–6.1 (6.5) µm in diam. (*n* = 35) including ornamentation, finely warted, hyaline to light green or light yellow, with a thick wall (≈0.7–1 µm), and a central oil droplet. Some spores with a short apiculus. Capillitium hyphae (2.5) 3–5 (up to 6) µm in diam. (walls ≈ 0.7–1 µm), yellow to hyaline, with pores.

Under SEM, spores are covered with ornamentation of conical to irregularly shaped warts. Some warts appear sharper, while others have more rounded apices and ridges; also, some warts are merged together to form irregularly shaped complexes. Apiculus short ≈0.7 µm, with a terminal opening.

Habitat: Xerothermophilous species, preferring dry continental regions, can be found in forest edges, grasslands and even sand steppe communities.

General distribution: Europe: Germany, Hungary, Italy, Romania, Russian Federation, Slovenia. Middle East: Israel. North America: USA. Central America: Costa Rica, Cuba (Caribbean Sea). Australasia: Australia, New Zealand.

Material examined: Israel. UJ. North of Almagor. On the ground. 23 December 2002. *Leg*. Y. Ur, *det*. M. Krakhmalnyi (HAI-G-78).

Notes: Spores of the current specimen correspond well with illustrations and descriptions presented by Rimóczi et al. [65] and Bates [46], although are, on average, 1 µm larger.

***Calvatia gigantea*** (Batsch) Lloyd, Mycol. Writ. 1(16): 166 (1904)

Basionym: *Lycoperdon giganteum* Batsch, Elench. fung., cont. prim. (Halle): 237 (1786)

Synonyms: *Bovista gigantea* (Batsch) Gray, Nat. Arr. Brit. Pl. (London) 1: 583 (1821); *Langermannia gigantea* (Batsch) Rostk., in Sturm, Deutschl. Fl., (Pilze Deutschl. 5–18) 3: 23 (1839); *Globaria gigantea* (Batsch) Quél., Mém. Soc. Émul. Montbéliard, Sér. 2 5: 370 (1873); *Calvatia gigantea* (Batsch) G. Cunn., Trans. Proc. N.Z. Inst. 57: 192 (1926); *Lasiosphaera gigantea* (Batsch) F. Šmarda, Fl. ČSR, B-1, Gasteromycetes: 308 (1958)

Habitat: Terricolous in meadows, parks and gardens, cultivated fields, on compost heaps, in rich deciduous forests and open woodland; in boreal to subtropical climates.

General distribution: Europe: Austria, Czech Republic, Denmark, Estonia, Finland, France, Germany, Greece, Hungary, Ireland, Italy, Lithuania, the Netherlands, Norway, Portugal, Romania, Russian Federation (European part), Slovakia, Spain, Sweden, Switzerland, United Kingdom. Mediterranean Islands: Balearic Islands (Spain), Corse (France). North America: Canada, Mexico, USA. Central America: Costa Rica. Middle East: Armenia, Georgia, Israel. Africa: South African Republic (possibly introduced). Arctic Territories: Iceland. Australasia: New Zealand.

Previous records from Israel: Binyamini reported the presence of the species, although the author did not provide information on studied specimen [55].

Notes: The species is easily recognizable due to its large size, ball-like appearance and rudimentary subgleba. It is the largest representative of the family Lycoperdaceae. Pegler et al. mentioned specimens exceeding 20 kg in weight [5]. The species was recorded in Israel by Binyamini [55]. The author presented one color photo of a gasterocarp and a short description without data on studied material. Some amateur mycologists have been reporting about specimens of *C. gigantea* found in the Golan Heights.

Genus: ***Disciseda*** Czern., Bull. Soc. Imp. Nat. Moscou 18(2, III): 153 (1845)
Type species: *Disciseda collabescens* Czern., Bull. Soc. Imp. Nat. Moscou 18: 153 (1845, as ‘*collabascens*’).
***Disciseda bovista*** (Klotzsch) Henn., Stud. Nat. Hist. Iowa Univ. 42: (128) (1903)

Basionym: *Geastrum bovista* Klotzsch [as ‘*Geaster*’], Fung. orb. terr. circumn. Meyen. coll.: 243 (1843)

Synonyms: *Disciseda bovista* (Klotzsch) Kambly, in Kambly & Lee, Hedwigia 17: 153 (1936); *Disciseda bovista* (Klotzsch) Eyndh., Medded. Nedl. Mycol. Ver. 27: 10 (1942)

Habitat: Terricolous, thermophilous species found in xeric habitats, in temperate to subtropical climates.

General distribution: Europe: Austria, Czech Republic, Denmark, France, Germany, Hungary, Italy, the Netherlands, Norway, Romania, Russian Federation (European part), Slovakia, Spain, Sweden, Switzerland. North America: USA. South America: Argentina. Middle East: Iran, Israel. Asia: Mongolia.

Previous records from Israel: Philistean Plain, Rehovot. On cultivated, brown-red sandy soil. 14 November 1957, 25 December 1957. Z. A. Hershenzon. [48,49].

Notes: The species was the first member of the family Lycoperdaceae found in Israel. It was recorded by Reichert and Avizohar-Hershenzon as *D. cervina* from the locality near Rehovot (PP) [48]. Dring and Rayss [49] presented a discussion on *D. cervina* and the validity of its earlier determination by Reichert and Avizohar-Hershenzon [48]. Trying to clarify this situation, the authors reexamined the material from Rehovot collected in 1957, and additionally studied type material of *D. cervina* from The Kew Herbarium. On the basis of macro- and micromorphological differences, Dring and Rayss [49] came to a conclusion that the material studied by Reichert and Avizohar-Hershenzon [48] appeared to present specimens of *D. bovista*.

**Figure 7:**
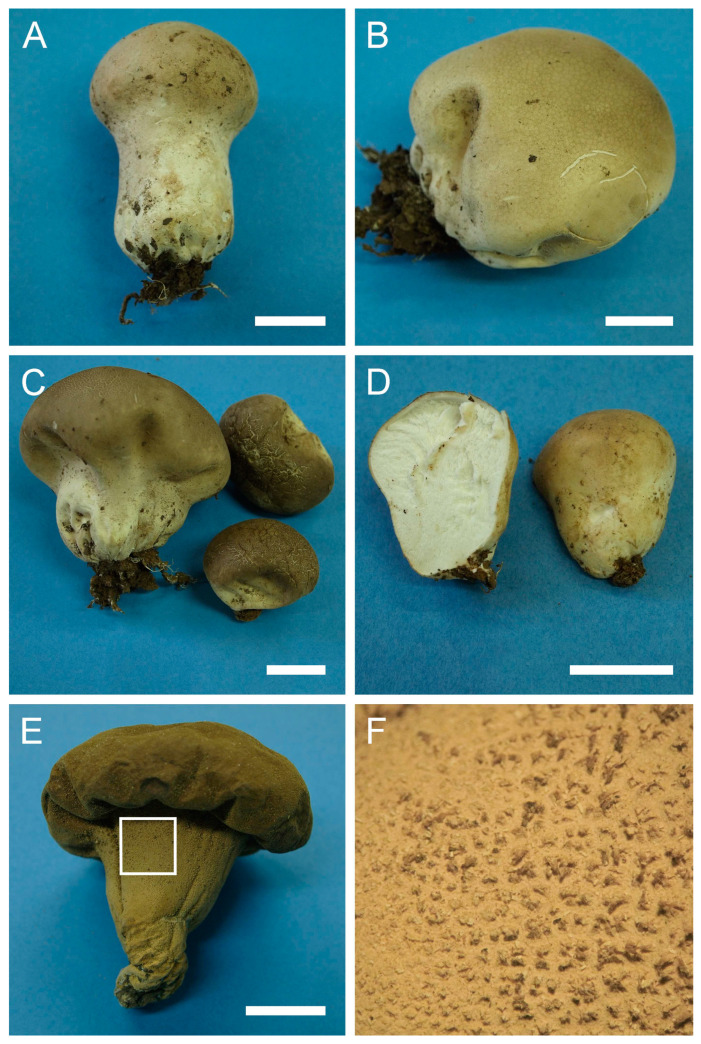
Different gasterocarp morphologies of *L. atropurpureum* (HAI-G-139, HAI-G-140, HAI-G-144, HAI-G-146, HAI-G-19). (**A**–**D**) fresh samples; (**E**) dried gasterocarp (scale bars = 2 cm). (**D**) Cross-section. (**F**) Exoperidium spines under stereo microscopy (zoomed from (**E**)).

**Figure 8:**
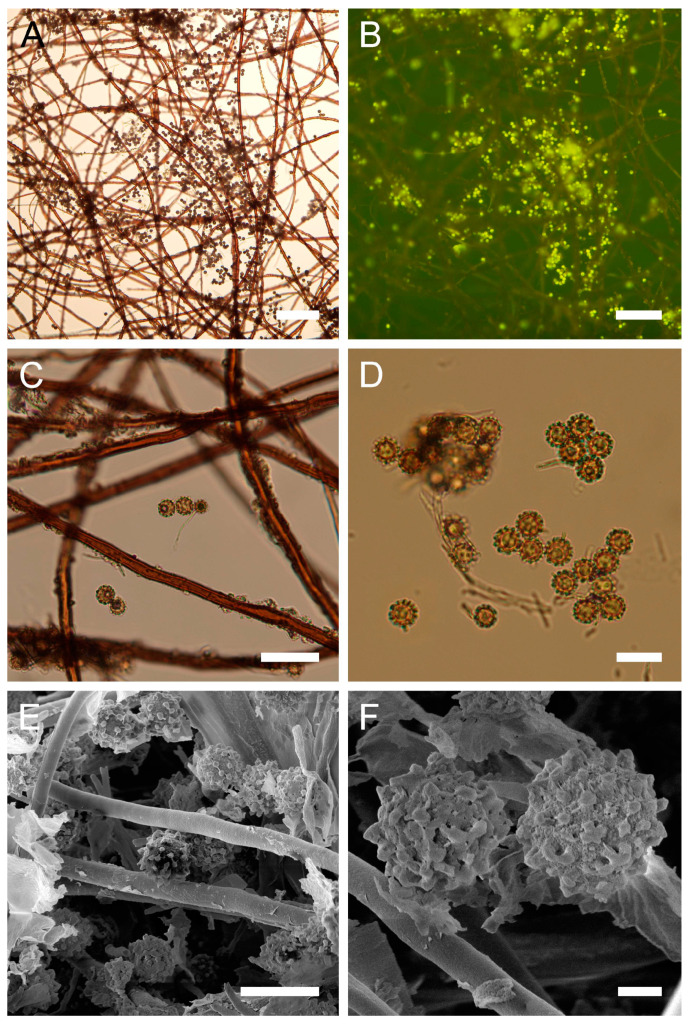
Micromorphology of *L. atropurpureum* (HAI-G-19, HAI-G-32). (**A**) General view of capillitial hyphae under light microscopy (scale bar = 100 µm). (**B**) Same picture taken in epifluorescence mode (scale bar = 100 µm). (**C**) Basidiospores and capillitium hyphae under light microscopy (scale bar = 20 µm). (**D**) Basidiospores under light microscopy (scale bar = 10 µm). (**E**) Basidiospores and capillitium hyphae under SEM (scale bar = 10 µm). (**F**) Microstructure of basidiospores under SEM (scale bar = 2 µm).

Genus: ***Lycoperdon*** Pers., Ann. Bot. (Usteri) 1: 4 (1794)
Type species: *Lycoperdon perlatum* Pers.: Pers., Observ. Mycol. (Lipsiae) 1: 4 (1796)
***Lycoperdon atropurpureum*** Vittad., in Vittadini C. Monogr. Lycoperd., Mem. Accad. Torino, vol. 2: 42 (1842). Figure 7, Figure 8 and Figure A1E.

Synonyms: *Lycoperdon atropurpureum* Vittad., in Vittadini C. Monogr. Lycoperd., Mem. Accad. Torino, vol. 2: 42 (1842) var. *atropurpureum*; *Lycoperdon atropurpureum* var. *catinense* Scalia, Atti Accad. Giorn. di Sci. Natur., Catania, IV 13: 24 (1900); *Lycoperdon stellare* (Peck) Lloyd, Mycol. Writ. 2 (Letter 20): 225 (1905); *Lycoperdon hirtum* var. *stellare* (Peck) Sacc. & Traverso, Syll. fung. (Abellini) 19: 1152 (1910); *Lycoperdon molle* var. *atropurpureum* (Vittad.) F. Šmarda, Fl. ČSR, B-1, Gasteromycetes: 350 (1958); *Lycoperdon molle* var. *hirtellum* (Peck) Kreisel, Feddes Repert. 64: 155 (1962); *Lycoperdon molle* var. *stellare* (Peck) Demoulin, Beih., Sydowia 8: 141 (1979).

Description: Gasterocarps commonly pestle-shaped with well-developed pseudostipe, sometimes subglobose-turbinate, tapering towards the base with wrinkles and folds. Young fruit bodies almost subglobose-sessile, with underdeveloped pseudostipe; during maturation, pseudostipe becomes larger and comprises up to 2/3 of gasterocarps’ length, with white branching mycelium (1) 2–2.5 cm long at the base, binding soil particles. Pestle-shaped specimens have the following dimensions: “cap” from 4–4.5 to 6.5–7 cm diam., 2.5–3 cm in height, pseudostipe (2.5) 3–3.5 (4) cm in diam., (3) 3.5–4 (4.5) in length, with full length of gasterocarp 5.5–7.5 cm. Subglobose-turbinate and depressed sessile specimens from 2.5–3 × 2.5–3 cm to (6) 7– 7.5 (8.5) cm in width, (5) 5.5–6.5 (7) cm in height. Color of pseudostipe from 3C, 6F to 52 (Buff), upper part 52 (Buff), 28 (Milky coffee), 27 Hazel, 17 (Snuff brown) to 12 (Fulvous). Exoperidium ornamented with fine dense spines (sometimes almost smooth), usually with a more abundant pattern of fine spines (often with connected tips) towards the base of the gasterocarp. Endoperidium white when young, then becoming yellow to cream-colored. Gleba, first white, in mature specimens—62 (Olivaceous) to 27 (Hazel). Subgleba strongly developed, white in young specimens, becoming 6F to chocolate brown.

Spores (5.5) 5.9–7 (8) μm in diam. (n = 36) including ornamentation, globose to subglobose, strongly warted, detached sterigmal remnants (5) 7–16 (up to 20–25) μm in length and ≈1 μm wide abundant in mounts. Some spores with a long pedicel attached. Capillitium of “*Lycoperdon*” type, brown, from 2–3 μm (wall 0.5 μm) to 4–5 μm (wall 1 μm) in width.

Under SEM, spores are strongly warted with cylindrical, conical or somewhat “phalliform” and irregularly shaped processes. Some warts are merged together, connected with tips, or even form arcs resembling half of a toroidal loop.

Habitat: Thermophilous species, prefers woodlands, and is a characteristic species for the Mediterranean–sub-Mediterranean oakwoods.

General distribution: Europe: Austria, Bulgaria, France, Germany, Greece, Italy, Portugal, Romania, Spain, Sweden, United Kingdom. Asia: China, Pakistan. (Records of the species from America should be treated carefully and, most likely, represent similar taxa. V. Demoulin considers that *L. atropurpureum* does not occur in America and is replaced there by *L. mauryanum* Pat. ex Demoulin).

Material examined: Israel. GH. Forest Odem. On the ground, under oaks. 5 January 2002. *Leg*. Y. Ur, *det*. M. Krakhmalnyi (HAI-G-19). CM. Horshat HaArbaim. On the ground, under *Quercus* sp. 10 February 2001. *Leg*. P. Tovbin, *det*. M. Krakhmalnyi (HAI-G-23). GH. Near Buq’ata and Forest Odem. On the ground, under *Quercus* sp. 4 January 1995. *Leg*. E. Nevo, S.P. Wasser, *det*. M. Krakhmalnyi (HAI-G-26). GH. Forest Odem. On the ground. 12 December 2005. *Leg*. Y. Ur, *det*. M. Krakhmalnyi (HAI-G-29). GH. Forest Odem, Tal Kasaa. On the ground, near *Quercus* sp. 11 January 2001. *Leg*. I. Shams, *det*. M. Krakhmalnyi (HAI-G-30). HP. Near Hula. On the ground, under oaks. 12 December 2005. *Leg*. Y. Ur, *det*. M. Krakhmalnyi (HAI-G-31). UG. Forest Satsufa, Oranim, North Meron. On the ground. 12 February 2003. *Leg*. Y. Ur, *det*. M. Krakhmalnyi (HAI-G-32). UG. Mt. Meron National Park. On the ground, *Pinus* and *Quercus* forest. 21 December 1996. *Leg*. E. Nevo, *det*. M. Krakhmalnyi (HAI-G-62). CM. Mt. Carmel National Park, Nahal Nesher. On the ground. 20 February 2010. *Leg*. P. Tovbin, *det*. M. Krakhmalnyi (HAI-G-70). UG. Park Goren. On the ground, *Quercus* forest. 21 January 2012. *Leg*. Z. Shafranov, *det*. M. Krakhmalnyi (HAI-G-99). UG. Park Goren. On the ground, *Quercus* forest. 21 January 2012. *Leg*. Z. Shafranov, *det*. M. Krakhmalnyi (HAI-G-100).—. *Leg*.—, *det*. M. Krakhmalnyi (HAI-G-115). GH. Forest Odem. On the ground. 1 December 2012. *Leg*. Z. Shafranov, A. Biketova, *det*. M. Krakhmalnyi (HAI-G-125). GH. Forest Odem. On the ground. 1 December 2012. *Leg*. Z. Shafranov, A. Biketova, *det*. M. Krakhmalnyi (HAI-G-126). GH. Forest Odem. On the ground. 1 December 2012. *Leg*. Z. Shafranov, A. Biketova, *det*. M. Krakhmalnyi (HAI-G-127). GH. Forest Odem. On the ground. 1 December 2012. *Leg*. Z. Shafranov, A. Biketova, *det*. M. Krakhmalnyi (HAI-G-129). GH. Forest Odem. On the ground. 8 December 2012. *Leg*. Z. Shafranov, A. Biketova, *det*. M. Krakhmalnyi (HAI-G- 139). GH. Forest Odem. On the ground. 8 December 2012. *Leg*. Z. Shafranov, A. Biketova, *det*. M. Krakhmalnyi (HAI-G-140). GH. Forest Odem. On the ground. 8 December 2012. *Leg*. Z. Shafranov, A. Biketova, *det*. M. Krakhmalnyi (HAI-G-144). GH. Forest Odem. On the ground. 8 December 2012. *Leg*. Z. Shafranov, A. Biketova, *det*. M. Krakhmalnyi (HAI-G-149). GH. Forest Odem. On the ground. 8 December 2012. *Leg*. Z. Shafranov, A. Biketova, *det*. M. Krakhmalnyi (HAI-G-150). GH. Forest Odem. On the ground. 8 December 2012. *Leg*. Z. Shafranov, A. Biketova, *det*. M. Krakhmalnyi (HAI-G-152). UG. Forest Hanita, Park Goren. On the ground. 22 December 2012. *Leg*. Z. Shafranov, *det*. M. Krakhmalnyi (HAI-G-165). GH. Forest Odem. On the ground. 28 December 2012. *Leg*. Y. Cherniavsky, *det*. M. Krakhmalnyi (HAI-G-174). UG. Mt. Meron National Park. On the ground. 28 December 2012. *Leg*. Y. Cherniavsky, *det*. M. Krakhmalnyi (HAI-G-175). UG. Mt. Meron National Park. On the ground. 28 December 2012. *Leg*. Y. Cherniavsky, *det*. M. Krakhmalnyi (HAI-G-176).

Notes: The species is one of the two most commonly distributed members of the family Lycoperdaceae in Israel, along with *L. lividum*. *Lycoperdon atropurpureum* is morphologically similar to *L. molle* and *L. decipiens* (for a wider discussion see the “Notes” under *L. decipiens*). Most of the studied Israeli samples of *L. atropurpureum* do not possess the purplish color of gleba, which is one of the diagnostic characteristics of the species, and have pestle-shaped gasterocarps. Spores under SEM differ from current specimens of *L. decipiens* by the shape and density of warts—*L. atropurpureum* has a very dense ornamentation of cylindrical, somewhat “phalliform”, and irregularly shaped processes. The spore surface between the warts is much rougher and more rugged, probably, due to remains of the perisporium. Some spores from the current SEM examination show similarities with SEM micrographs of *L. decipiens* by Rimóczi et al. [65], while others have ornamentation of cylindrical warts with rounded tips, resembling spores’ micrographs of *L. molle* from the studies of Bates [46] and Bates et al. [43]. SEM photos of Moreno et al. display ornamentation of high (more than 1 μm) conical processes with sharp or rounded tips [89]. All of the abovementioned lead to the assumption that spore ornamentation of *L. atropurpureum* can demonstrate great variability in structure, density, and arrangement of warts.

**Figure 9:**
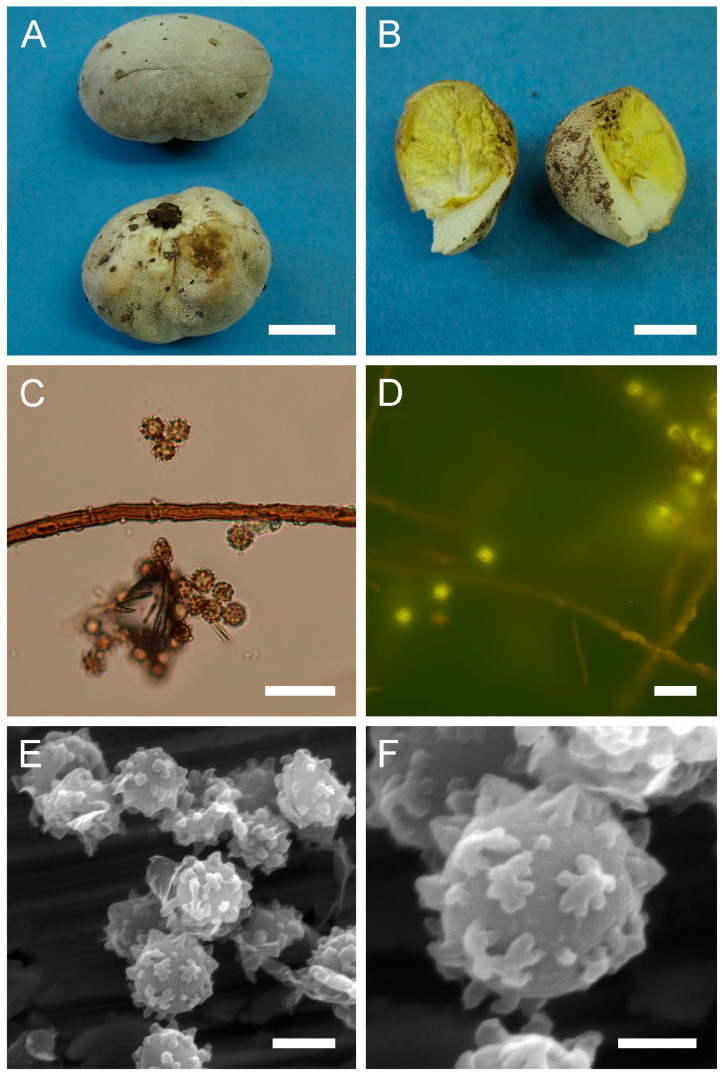
Macro- and micromorphology of *L. decipiens* (HAI-G-142, HAI-G-145). (**A**) Gasterocarp—top and bottom view (scale bar = 2 cm). (**B**) Cross-section of the gasterocarp (scale bar = 1 cm). (**C**) Basidiospores and capillitium hypha under light microscopy (scale bar = 20 µm). (**D**) Basidiospores and capillitium hyphae under light microscopy (epifluorescence mode, scale bar = 20 µm). (**E**) Basidiospores under SEM (scale bar = 5 μm). (**F**) Microstructure of the basidiospore under SEM (scale bar = 2 µm).

**Figure A2:**
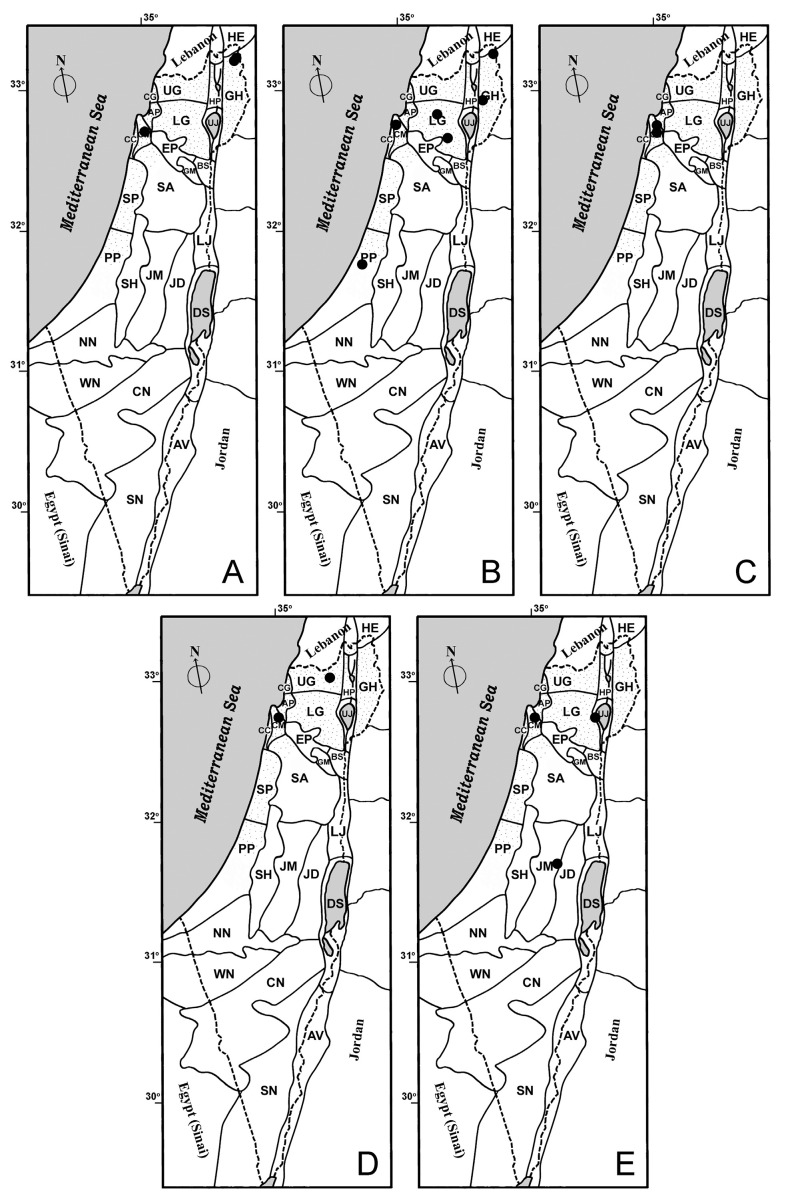
Distribution of Lycoperdaceae species in Israel. (**A**) *Lycoperdon decipiens*; (**B**) *L. lividum*; (**C**) *L. niveum*; (**D**) *L. perlatum*; (**E**) *L. pratense*.

***Lycoperdon decipiens*** Durieu & Mont., in Durieu, Expl. Sci. Algérie, Bot. 1. Crypt.: 380 (1848) [1846–49]. Figure 9 and Figure A2A.

Synonyms: *Bovista cepiformis* Wallr., Fl. crypt. Germ. (Norimbergae) 1: no. 2253 (1831); *Lycoperdon decipiens* Durieu & Mont., in Durieu, Expl. Sci. Algerie. 1(livr. 10): 380 (1848) [1846–49] var. *decipiens*; *Lycoperdon cepiforme* (Wallr.) Bonord., Bot. Ztg. 17: 595 (1859); *Lycoperdon decipiens* var. *delicatum* F. Šmarda, Fl. ČSR, B-1, Gasteromycetes: 354 (1958)

Description: Gasterocarps subglobose to subglobose-turbinate, subglobose-sessile, compressed vertically, usually wider than high, ranging from 2 cm in width and 2–2.5 cm in height, to 5–6 cm in width and 3–3.5 in height, tapering towards the base with folds and wrinkles, without pseudostipe and with poorly developed subgleba, with 0.5–1 (up to 1.5) cm basal turf of mycelium binding substratum. Exoperidium almost smooth at the top, with minute, fragile spines 6F to 52 (Buff) more abundant near the base of the gasterocarp. Some mature specimens have cracks and wrinkles of exoperidium in the upper part. Color of the exoperidium from 52 (Buff) at the base, gradually becoming 32 (Clay buff) and 34 (Smoke grey) towards the top. In young specimen, gleba from 56 (Yellowish green) to 57 (Greenish yellow), becoming in mature specimens 61 (Grey olivaceous), 62 (Olivaceous), 16 (Cigar brown) to 27 (Hazel). Subgleba, first white, then becoming olivaceous to greyish brown. Spore deposit brown to greyish brown. Spores (4) 4.4–5.6 (6) μm in diam. (n=60) excluding ornamentation, globose to subglobose, strongly warted, yellow to light brown, mixed with sterigmal remnants. Some spores with pedicel attached up to 3 μm in length. Capillitium of “*Lycoperdon*” type, brown, fragile, from (2) 2.5–4 μm (wall 0.5–0.6) to 5–6 (up to 7) μm (wall 0.7–1 μm). Pores rarely seen ≈1 μm circular to ovoid in shape, usually in thin hyphae. Exoperidium of simple sphaerocysts.

Spores under SEM subglobose, strongly verrucose. Relatively dense ornamentation consisting of irregularly shaped verrucae, usually closer to the conical shape, was observed. Most of the warts merged together in groups, forming complexes with separated bases and connected tips, tending to be conical in shape. Some warts connected together by low thin anastomoses. Spore surface between warts appears almost smooth. Apiculus usually less than 1.5–2 μm in length with a terminal pore can be observed.

Habitat: Thermophilous species, can be found in dry, mostly calcareous, grasslands of the forest steppe and light deciduous forests.

General distribution: Europe: Andorra, Austria, Denmark, Germany, Great Britain (England and Ireland), Hungary, Slovenia, Spain, Sweden. Middle East: Israel. North America: Canada and USA.

Material examined: Israel. CM. Mt. Carmel National Park, “Little Switzerland”. On the ground. 21 February 2010. *Leg*. P. Tovbin, *det*. M. Krakhmalnyi (HAI-G-63). GH. Forest Odem. On the ground, open area. 6 November 2006. *Leg*. Y. Ur, *det*. M. Krakhmalnyi (HAI-G-75). GH. Forest Odem. On the ground. 8 December 2012. *Leg*. Z. Shafranov, A. Biketova, *det*. M. Krakhmalnyi (HAI-G-142). GH. Forest Odem. On the ground. 8 December 2012. *Leg*. Z. Shafranov, A. Biketova, *det*. M. Krakhmalnyi (HAI-G-145).

Notes: *Lycoperdon decipiens* can be easily confused with *L. atropurpureum* and *L. molle*. These three species form the so-called *Lycoperdon atropurpureum–molle–decipiens* species complex, because they show high similarity in macro- and micromorphological characteristics, and also turned out to be related in terms of molecular phylogenetics. Prior to the present study, only *L. atropurpureum* and *L. molle* were known for Israel; both were mentioned for the first time in the work of Binyamini [55].

All three species have thin exoperidium spines and show great variability in color, shape, and size of gasterocarps. Current specimens of *L. decipiens* have subglobose-turbinate gasterocarps, compressed vertically, while Israeli specimens of *L. atropurpureum* usually possess very well-developed pseudostipe and are pestle-shaped, resembling specimens of *Lycoperdon excipuliformis* (= *Handkea excipuliformis*).

Current SEM photos differ from the specimens examined by Rimóczi et al. [65], which had denser ornamentation of cylindrical, conical, and somewhat “phalliform” processes with remains of the perisporium, covering regions of the spore surface. Spores in the present study have less dense ornamentation of conical processes, which tend to merge together in groups, forming conical structures with separated bases and connected tips.

**Figure 10:**
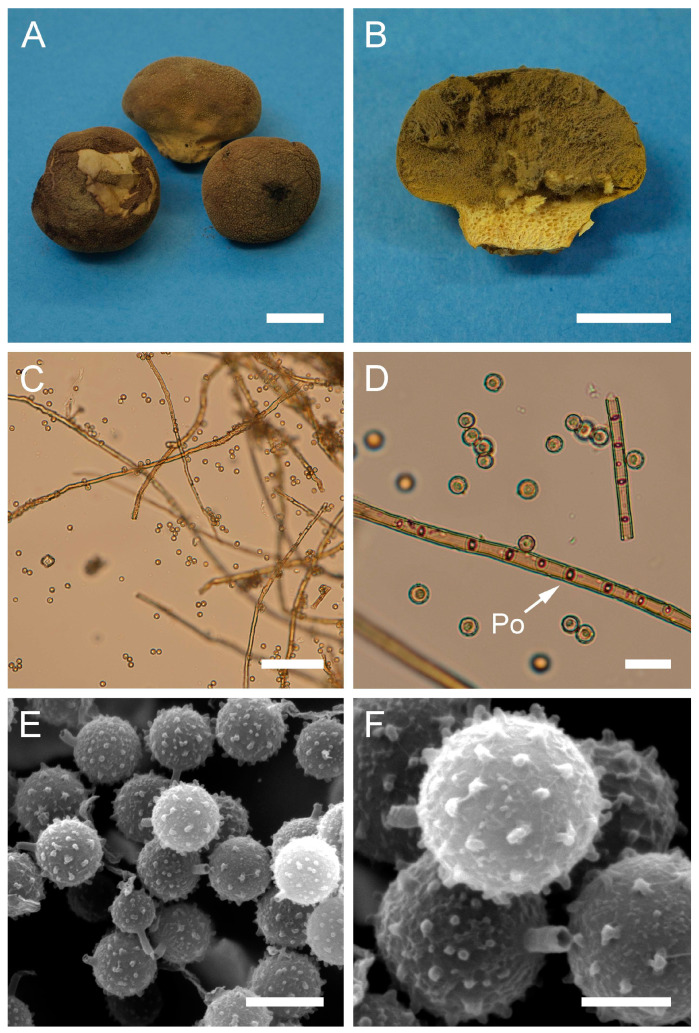
Macro- and micromorphology of *L. lividum* (HAI-G-66). (**A**) Gasterocarps (scale bar = 1 cm). (**B**) Cross-section of the gasterocarp (scale bar = 1 cm). (**C**) Basidiospores and capillitium hyphae under light microscopy (scale bar = 40 µm). (**D**) Basidiospores and hyphae under light microscopy; Po—pores (scale bar = 10 µm). (**E**) Basidiospores under SEM (scale bar = 5 µm). (**F**) Microstructure of basidiospores under SEM (scale bar = 2 µm).

***Lycoperdon lividum*** Pers., J. Bot. (Desvaux) 2: 18 (1809). Figure 10 and Figure A2B.

Synonyms: *Lycoperdon cervinum* Bolton, Hist. fung. Halifax (Huddersfield) 3: 116, Table 116 (1790) [1789]; *Lycoperdon cookei* Massee, J. Roy. Microscop. Soc.: 14 (1887); *Lycoperdon spadiceum* Pers., J. Bot. (Desvaux) 2: 20 (1809)

Description: Gasterocarps subglobose, compressed at the top, 2.5–3.5 cm in height and 2–2.5 cm in width, tapering towards the base with folds and wrinkles, and with a compact subgleba. Some gasterocarps separated from the mycelium, while others bear rhizomorphs at the base ≈1 cm in length, intermixed with soil particles. Color at the base from 52 (Buff) to 6F, becoming darker towards the top—27 (Hazel) to 17 (Snuff brown). Exoperidium almost smooth at the base, and with minute warts (somewhat granulate), cracks and wrinkles at the top. After maturation, exoperidium disintegrates, revealing a papery endoperidium 52 (Buff), which in some specimens tears irregularly at the top, while others have a well-defined ostiole. Ostiole, at first, appears as a dark spot on the exoperidium, and after sloughing of the latter, becomes more or less regular in shape opening. Gleba 62 (Olivaceous) to 27 (Hazel), subgleba compact, alveolate, light yellow with pinkish tint (4D). Spore print olivaceous-yellow. Spores (3.5) 3.8–4.6 (5) μm in diam. (n=44) excluding ornamentation, globose to subglobose, asperulate to finely verrucose, light yellow, with an oil droplet, sometimes with short pedicel attached (≈1–1.5 µm long). Capillitium of “*Lycoperdon*” type, yellowish, (2.5) 3–6 (up to 7) µm in width, walls from 0.3–0.4 to 0.6–0,7 μm (rarely reaching up to 1 µm). Pores in abundance, circular and elliptical in shape. Exoperidium comprised of subglobose to ovoid sphaerocysts 30–35 × 20–30 µm.

Under SEM, spores globose, covered with conical or irregularly shaped processes with rounded apices. Some warts merged together, or have connected tips, rarely connected together by low meshes. Spore surface between the warts covered with low, irregularly shaped (somewhat granulate) verrucae. Apiculus 0.7–1 (up to 2) µm in length with a terminal pore is observed.

Habitat: Terricolous and found on weakly acid to calcareous humus or soil in dry grassland sites, steppes, pastures, meadows, dry lawns, also on sandy soils, dunes, limestone, and gypsum; in subarctic to subtropical climates.

General distribution: Europe: Austria, Belgium, Bulgaria, Czech Republic, Denmark, Estonia, Finland, France, Germany, Greece, Hungary, Ireland, Italy, Lithuania, Netherlands, Norway, Poland, Portugal, Romania, Russian Federation (European Part), Slovakia, Spain, Sweden, Switzerland, United Kingdom, former Yugoslavia. Mediterranean Islands: Balearic Islands (Spain), Corse (France). Macaronesia: Canary Islands (Spain). Arctic Territories: Greenland, Iceland, Svalbard (Norway). Middle East: Armenia, Israel. Asia: Japan. North America: USA. Australasia: Australia, New Zealand.

Material examined: Israel. GH. Katzrin. On the ground, under pine trees. 3 March 2003. *Leg.* Yair Ur, *det.* M. Krakhmalnyi (HAI-G-10). CM. Cherion Shar Ha-Carmel. On the ground, near *Pinus brutia*. 3 February 2001. *Leg.* I. Baskin, *det.* M. Krakhmalnyi (HAI-G-15). GH. Masaade, Kadzrin forest. On the ground, near pines. 21 January 2002. *Leg.* Yair Ur, *det.* M. Krakhmalnyi (HAI-G-66). PP. Hatzor. On the ground. 30 March 2003. *Leg.* Yair Ur, *det.* M. Krakhmalnyi (HAI-G-71). LG. Beit Keshet forest. On the ground, under oak. 17 February 2004. *Leg.* Yair Ur, *det.* M. Krakhmalnyi (HAI-G-76). UG. Mt. Hazon, Hazon forest. On the ground, under oaks. 1 February 2004. *Leg.* Yair Ur, *det.* M. Krakhmalnyi (HAI-G-77).

Previous records from Israel: Judean Mountains, near Jerusalem. 25 January 1952. S. Borut (Kew). Ibid., (no date), (Kew). Judean Mountains, Jerusalem. March 1953 (Kew). Ibid. 9 January 1957 (Kew). Ibid. 21 January 1958 (Kew). Ibid. Zoological Garden. December 1955 (Kew). Ibid. 9 January 1957 (Kew). Ibid. On lawn. 9 January 1957 (Kew). Judean Mountains, Qiryath-Anavim. 2 March 1957 (Kew). Judean Mountains, Nes-Harim. 11 February 1959 (Kew). Judean Mountains, Bab-el-waad. 11 March 1959 (Kew) [49].

Notes: *Lycoperdon lividum* is a small puffball characterized by a granulose exoperidium, asperulate basidiospores and a fragile capillitium with pores of various shapes. The species was recorded in Israel by Dring and Rayss as *L. spadiceum* Schaeff. from multiple localities in the Judean Mountains [49]. Later, the presence of the species was reported by Binyamini [55].

SEM photos from the current examination show a high similarity with SEM micrographs of *L. lividum* specimens from Hungary, provided in the study of Rimóczi et al. [65], in the structure of spore ornamentation, with the exception that in the present study the spore surface between the warts is covered with denser and larger verrucae. SEM photo of “*L.* cf. *dermoxanthum*” presented by Bates et al. [43] shows denser ornamentation of rounded warts and almost smooth spore wall surface between them (for a wider discussion regarding “*L.* cf. *dermoxanthum*” [43] and *L. lividum*, see “Molecular phylogeny of Lycoperdaceae ” section).

***Lycoperdon molle*** Pers., Syn. meth. fung. (Göttingen) 1: 150 (1801)

Synonyms: *Lycoperdon molle* Pers., Syn. meth. fung. (Göttingen) 1: 150 (1801) var. *molle*; *Lycoperdon gemmatum* var. *furfuraceum* Fr., Syst. mycol. (Lundae) 3(1): 38 (1829); *Lycoperdon gemmatum* var. *molle* (Pers.) De Toni, Syll. fung. (Abellini) 7: 107 (1888)

Habitat: Terricolous on neutral to calcareous soils in deciduous, or mixed coniferous and deciduous woodland, in subarctic to subtropical climates.

General distribution: Europe: Austria, Belgium, Czech Republic, Denmark, Estonia, Finland, France, Germany, Greece, Hungary, Ireland, Italy, Lithuania, the Netherlands, Norway, Poland, Portugal, Romania, Slovakia, Sweden, Switzerland, United Kingdom, former Yugoslavia. Mediterranean Islands: Balearic Islands (Spain), Corse (France). Arctic Territories: Iceland, Svalbard (Norway). Middle East: Iran, Israel. North America: USA. Asia: Mongolia, Japan.

Previous records from Israel: The species was mentioned in the monograph of Binyamini [55]. The author presented one color photo of gasterocarps and a short description without providing some basic information about studied specimens.

Notes: The species is characterized by turbinate to pyriform gasterocarps, exoperidium typically covered with short delicate spines, densely verrucose spores intermixed with sterigmal remnants, and thick-walled capillitium with abundant small pores. *Lycoperdon molle* can be confused with similar species, including *L. atropurpureum* and *L. decipiens*, both present in Israel (for a wider discussion see “Notes” under the listed species).

**Figure 11:**
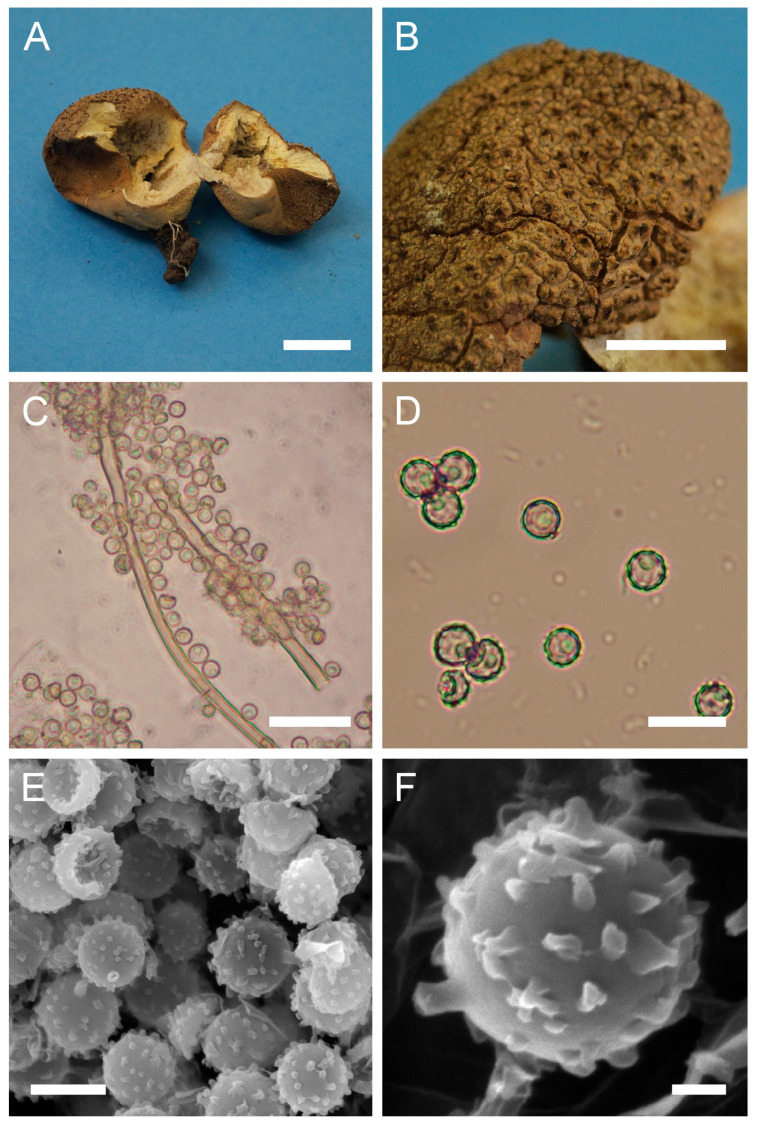
Macro- and micromorphology of *L. niveum* (HAI-G-72). (**A**) Cross-section of the gasterocarp (scale bar = 1 cm). (**B**) Structure of exoperidium (scale bar = 5 mm). (**C**) Basidiospores and capillitium hyphae under light microscopy (scale bar = 20 µm). (**D**) Basidiospores under light microscopy (scale bar = 10 µm). (**E**) Basidiospores under SEM (scale bar = 5 µm). (**F**) Microstructure of the basidiospore under SEM (scale bar = 1 µm).

***Lycoperdon niveum*** Kreisel, Khumbu Himal 6(1): 30 (1969). Figure 11 and Figure A2C.

Description: Gasterocarps 3 cm in width and 3 cm in height, subglobose-turbinate, tapering towards the base, with a compact subgleba and 1–1.5 cm long strands of basal mycelium, mixed with soil particles. Exoperidium from 52 (Buff) at the base to light brown towards the top. The upper part of the gasterocarp is covered with short dark brown spines with connected tips, and cracks, forming some kind of a reticulum, while near the base small fragile light-colored spines were observed. Gleba, first 3C—5E, then 27 (Hazel), 61 (Grey olivaceous), and 62 (Olivaceous). Subgleba compact, poroid, 4D. Spores globose, (4) 4.1–5.4 (6) μm in diam. (n = 41) (wall ≈0.5 μm thick) excluding ornamentation, distinguishably verrucose, hyaline to light green, with an oil droplet, sometimes with short pedicel attached (usually less than 1–1.5 μm). Capillitium of “*Lycoperdon*” type, from 2–2.5 (wall ≈0.2–0.3 μm) to 3.5–5 μm (wall ≈0.7–1 μm), hyaline to light green-yellow. Pores not observed.

Spores under SEM globose, with ornamentation of conical processes and short warts. Some processes connected by tips and form complexes. Additionally, some verrucae connected together by low, thin anastomoses. Spore surface between verrucae appears smooth. Apiculus usually around 0.7 μm in length with a terminal pore is present.

General distribution: Asia: China? Nepal. Europe: Norway, Republic of Macedonia, Spain, Sweden. Middle East: Israel. Arctic Territories: Iceland.

Material examined: Israel. CM. Mt. Carmel National Park, Nahal Nesher. On the ground. 21 January 2006. *Leg*. Y. Ur, *det*. M. Krakhmalnyi (HAI-G-72). CM. Horshat HaArbaim. On the ground, under *Quercus* sp. 7 February 2001. *Leg*. P. Tovbin, *det*. M. Krakhmalnyi (HAI-G-74).

Notes: Kreisel described *L. niveum* from material collected in the Himalayas (Nepal) [80]. The species was subsequently reported from Iceland and Norway. The current finding of *L. niveum* is the first record for the territory of the Middle East, and for Israel in particular. It is registered from two localities, both on the Carmel Mountain.

**Figure 12:**
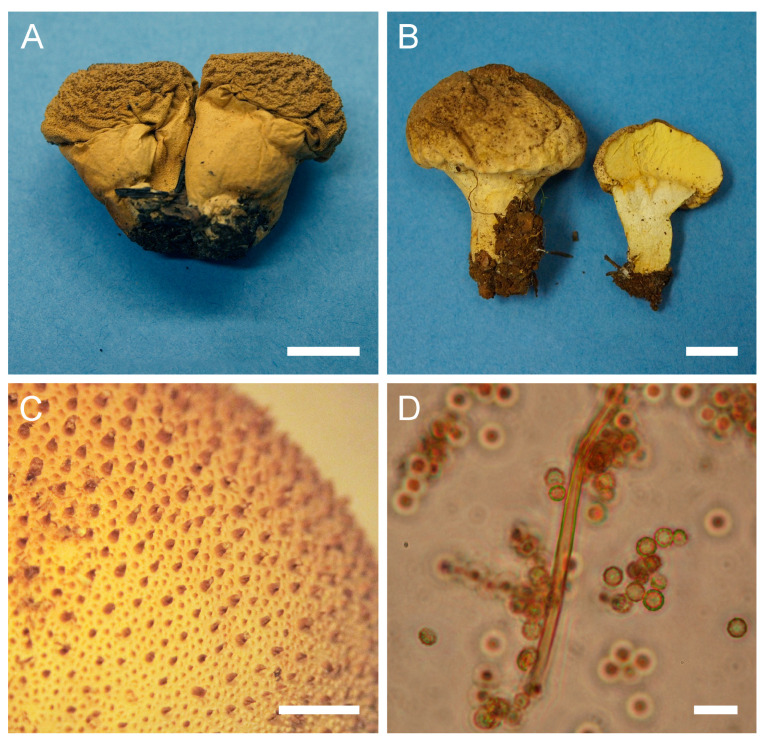
Macro- and micromorphology of *L. perlatum* (HAI-G-69, HAI-G-171). (**A**,**B**) Dried gasterocarps (scale bar = 1 cm). (**C**) Exoperidial spines under stereo microscopy (scale bar = 5 mm). (**D**) Basidiospores and capillitium hyphae under light microscopy (scale bar = 10 µm).

**Figure 13:**
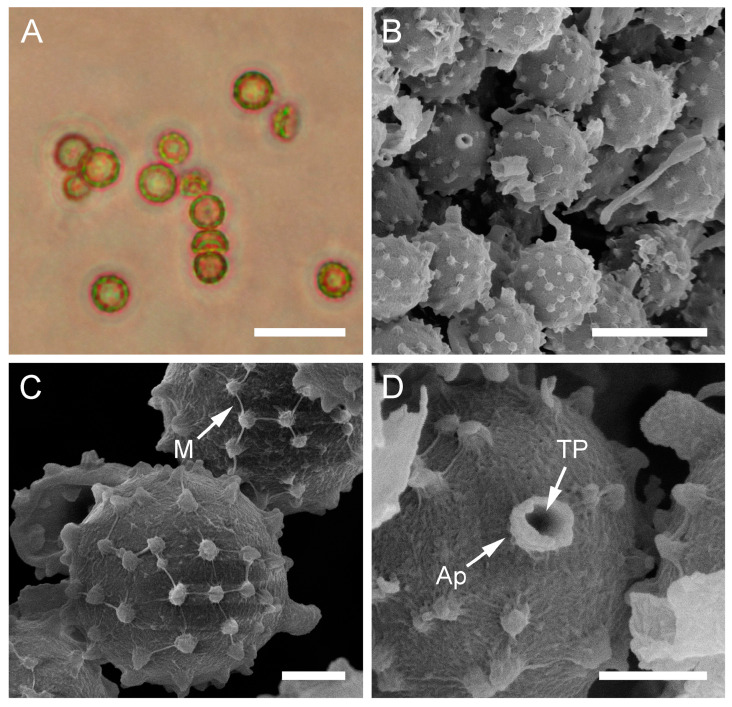
Micromorphology of *L. perlatum* basidiospores (HAI-G-171). (**A**) Basidiospores under light microscopy (scale bar = 10 µm). (**B**) Basidiospores under SEM (scale bar = 5 µm). (**C**) Basidiospores with clearly visible conical ornamentation and meshes (M) (scale bar = 1 µm). (**D**) Apiculus (Ap) with a terminal pore (TP) (scale bar = 1 µm).

***Lycoperdon perlatum*** Pers.: Pers. Syn. Meth. Fung.: 145 (1801). Figure 12, Figure 13 and Figure A2D.

Basionym: *Lycoperdon perlatum* Pers., Observ. Mycol. (Lipsiae) 1: 4 (1796)

Synonyms: *Lycoperdon lacunosum* Bull., Herb. Fr. 2: Table 52 (1782) [1781–82]; *Lycoperdon gemmatum* Batsch, Elench. fung., cont. prim. (Halle): 147 (1783); *Lycoperdon perlatum* var. *albidum* Alb. & Schwein., Consp. fung. (Leipzig): 80 (1805); *Lycoperdon gemmatum* var. *perlatum* (Pers.) Fr., Syst. Mycol. (Lundae) 3(1): 37 (1829); *Lycoperdon bonordenii* Massee, J. Roy. Microscop. Soc.: 713 (1887); *Lycoperdon perlatum* var. *lacunosum* (Bull.) Rea, Brit. basidiomyc. (Cambridge): 34 (1922); *Lycoperdon perlatum* var. *bonordenii* (Massee) Perdeck, Blumea 6: 505 (1950); *Lycoperdon perlatum* var. *dobremezianum* Kreisel, Beih. Feddes Repert. Spec. Nov., Beih. 87(1–2): 103 (1976)

Description: Gasterocarps solitary or in small groups, turbinate to pestle-shaped, 2.5–4.5 cm in height and 2–4 cm in width, with a strongly developed sterile subgleba. Pseudostipe from 1/2 reaching to 2/3 of gasterocarp’s height, either cylindrical and even throughout its length, or tapering towards the base. Rhizoids 0.5–1 cm (up to 2 cm) long, binding substrate. Exoperidium 5E to 52 (Buff) at the base, gradually becoming darker towards the top—27 (Hazel) to 17 (Snuff brown), with the darkest spot on the place of ostiole. Exoperidium in the apical portion of the gasterocarp bears 0.5–1 mm long conical spines 17 (Snuff brown) to 24 (Date brown), which do not coalesce, each surrounded by a circular row of smaller warts. After gasterocarp’s maturation, large spines wear off, leaving scars surrounded by warts, that produce a characteristic well-defined reticulate pattern on the endoperidium. Down towards the pseudostipe, a pattern of fine even warts 5E to 52 (Buff) was observed; their abundance decreased from top to bottom. Some specimens with almost smooth pseudostipe, free of any ornamentation. The apex of immature gasterocarps bears a prominence or papilla, characterized by darker coloring, after maturation becoming an apical ostiole, with orbicular to irregularly shaped opening. Endoperidium, first yellow 6F, then becoming papery and shiny olive-grey, olive-brown. Gleba, first white or light yellow 6F, 8G, 50 (Straw), becoming olive-brown to grey-brown. Subgleba strongly developed, alveolate, first white to 6F, 8G, then olive-brown to brown. Pseudocolumella present. Spore deposit yellow-brown, olive-brown to grey-brown.

Basidiospores under light microscopy (3) 3.6–4.3 (4.5) µm in diam. (n = 35) excluding ornamentation, hyaline to light green-yellow, sometimes with a reddish tint, globose to subglobose, verrucose, with short pedicel ≈0.5–1 µm (up to 3 µm). Sterigmal remnants occasionally present in mounts. Spore wall 0.5–0.7 µm. Usually a central oil droplet can be observed. Capillitium of the “*Lycoperdon*” type, elastic, yellow to yellow-brown, 3–6 µm wide, with relatively thin walls. Pores rare. Exoperidium consisting of sphaerocysts.

Under SEM, spores globose to slightly subglobose, showing distinct ornamentation of conical processes, more or less equal in size (about 0.3 µm), with either sharper, or with rounded and flattened apices. The ornamentation irregularly dispersed. Most of the warts connected together by low, thin meshes, forming a somewhat reticulated pattern. Some warts merged together, or occasionally connected by tips. The spore surface between warts and meshes not perfectly smooth, and appears rough and rugged. The apiculus 0.5–0.7 µm long, with a terminal pore 0.3–0.5 µm wide, is observed.

Habitat: Occurs in deciduous and coniferous woods, cosmopolitan species present in all continents, except Antarctica.

General distribution: Europe: Andorra, Austria, Czech Republic, Denmark, Estonia, Germany, France, Ireland, Italy, Latvia, Luxembourg, Norway, Portugal, Romania, Russian Federation (Central European part), Slovakia, Slovenia, Spain, Sweden, United Kingdom. Mediterranean Islands: Cyprus. Middle East: Israel. Africa: Kenya, Nigeria. Asia: Mongolia, Japan. Arctic Territories: Iceland. North America: Canada, Mexico, USA. Central America: Costa Rica. South America: Argentina. Australasia: Australia, New Zealand.

Material examined: Israel. CM. Mt. Carmel National Park, Nahal Nesher. On the ground. 28 December 2004. *Leg*. I. Tovbina, *det*. M. Krakhmalnyi (HAI-G-69). UG. Mt. Meron National Park. On the ground, under oak. 7 February 2004. *Leg*. Y. Ur, *det*. M. Krakhmalnyi (HAI-G-80). UG. Mt. Meron National Park. On the ground. 26 December 2012. *Leg*. Y. Cherniavsky, *det*. M. Krakhmalnyi (HAI-G-171). UG. Mt. Meron National Park. On the ground. 28 December 2012. *Leg*. Y. Cherniavsky, *det*. M. Krakhmalnyi (HAI-G-173).

Notes: Cosmopolitan species found in all continents, except Antarctica. Taking into consideration its wide distribution, it is not surprising that *L. perlatum* is now registered for Israel. Some amateur mycologists and mushroom hunters reported its presence in the country, but this could not be proven until the material previously stored in the HAI Herbarium and collected during additional field trips was studied. Currently, *L. perlatum* is known from two localities—Mt. Carmel and Mt. Meron.

The type species of the genus *Lycoperdon* is fairly easy to distinguish by its pestle-shape and reticulate pattern of exoperidium with large spines, encircled by small warts. In the current samples, gasterocarps with two pseudostipe morphologies were observed. Samples HAI-G-69 and HAI-G-80 have almost cylindrical, wide pseudostipe (≈80% of gasterocarp’s width), with very little tapering towards the base. Moreover, this type of pseudostipe is almost free of any warts. Gasterocarps of the second type (HAI-G-171, HAI-G-173) have a notably thinner and slender pseudostipe, which shows a very strong tapering towards the base (it can constitute less than 35–40% of gasterocarp’s width), and bears an ornamentation of fine even warts.

A molecular phylogenetic study carried out by Larsson and Jeppson demonstrated that *L. perlatum* clusters with *L. marginatum* and *L. norvegicum*, along with some species of *Morganella* and *Vascellum* in a clade with 59 BS/1.00 BPP support [41]. The current analysis recovered the same topology of the species.

Bates [46] and Bates et al. [43] presented high-quality SEM micrographs of investigated representatives of the family Lycoperdaceae. Examination and a comparison of *L. perlatum* spores’ micrographs, showed that spores of current specimens are very similar in terms of size and shape, and also have a distinct ornamentation of conical processes, yet differ in some aspects. The number of meshes between warts in Bates et al. [43] specimens is much lower than in current samples, and additionally the spore surface between warts and meshes appears smoother compared to Israeli material.

**Figure 14:**
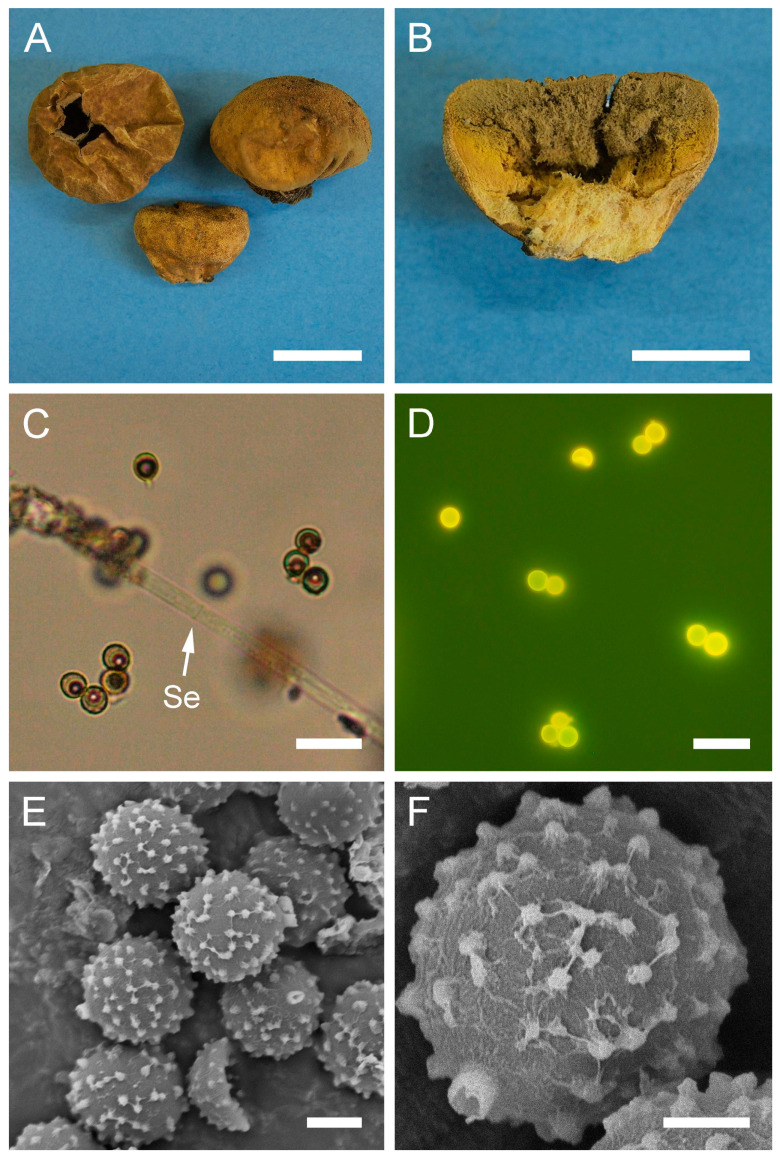
Macro- and micromorphology of *L. pratense* (HAI-G-105). (**A**) Dried gasterocarps (scale bar = 2 cm). (**B**) Cross-section of the gasterocarp (scale bar = 1 cm). (**C**) Basidiospores and paracapillitium under light microscopy; Se—septum (scale bar = 10 µm). (**D**) Basidiospores under light microscopy (epifluorescence mode, scale bar = 10 µm) (**E**) Basidiospores under SEM (scale bar = 2 µm). (**F**) Microstructure of the basidiospore under SEM (scale bar = 1 µm).

***Lycoperdon pratense*** Pers., Neues Mag. Bot. 1: 87 (1794). Figure 14 and Figure A2E.

Synonyms: *Vascellum pratense* (Pers.) Kreisel, Feddes Repert. Spec. Nov. 64: 159 (1962); *Lycoperdon hiemale* Bull., Herb. Fr. 2: 148 (1782) [1781–82]; *Utraria pratensis* (Pers.) Quél., Mém. Soc. Émul. Montbéliard, Sér. 2 5: 368 (1873); *Lycoperdon depressum* Bonord., Bot. Zeitung 15: 611 (1857); *Lycoperdon subpratense* Lloyd, Mycol. Writ. 2(Letter 20): 231 (1905); *Calvatia subpratensis* (Lloyd) Coker & Zeller, Mycologia 39(3): 305 (1947); *Vascellum depressum* (Bonord.) F. Šmarda, Bull. int. Acad. pol. Sci. Lett. 1: 305 (1958); *Vascellum pratense* subsp. *subpratense* (Lloyd) Kreisel, Feddes Repert. Spec. Nov. Regni Veg. 68: 87 (1963); *Calvatia depressa* (Bonord.) Zeller & A.H. Sm., Lloydia 27: 171 (1964); *Vascellum subpratense* (Lloyd) P. Ponce de León, Fieldiana, Bot. 32(9): 113 (1970)

Description: Gasterocarps in small groups, turbinate, compressed from the top, 2.5–4 cm in height and 2.5–3 cm in width, with wide soil bulb (1.5 cm in width and 0.7–1 cm in length). Exoperidium 52 (Buff) to 5E, bearing minute spines 27 (Hazel) with coalescing tips in the upper portion of gasterocarp. Endoperidium almost smooth, papery, lighter than exoperidium. Gasterocarps open with irregular terminal tear ≈1 × 0.7 cm. Gleba of mature specimens from 8G, 9H to 61 (Grey olivaceous), 17 (Snuff brown) and 27 (Hazel). Diaphragm distinct. Subgleba comprising one-third of the gasterocarp, alveolate, 5E to 4D. Spore print 17 (Snuff brown) to 27 (Hazel). Basidiospores (3) 3.4–4.2 (4.7) µm (n = 46) excluding ornamentation, globose to subglobose, somewhat ovoid, finery verrucose, hyaline to light green or light yellow, with an oil droplet, and with a short ≈0.5–0.7 µm pedicel attached. Occasionally, sterigmal remnants up to 10–15 µm long can be observed in mounts. Paracapillitium thin-walled, septate, non-poroid, hyaline, 2.5–5.5 µm in width.

Habitat: Terricolous in grassland including fields, meadows, pastures, garden lawns, roadsides, open woodland, also amongst short turf on heathland and downland, nitrophilous; temperate to subtropical (probably introduced in tropical and southern subtropical climates).

General distribution: Europe: Austria, Belgium, Bulgaria, Czech Republic, Denmark, Estonia, Finland, France, Germany, Greece, Ireland, Lithuania, Netherlands, Norway, Poland, Portugal, Romania, Russian Federation (European part), Slovakia, Sweden, Switzerland, United Kingdom. Mediterranean Islands: Balearic Islands (Spain), Corse (France). Macaronesia: Azores (Portugal), Cape Verde, Canary Islands (Spain). Africa: Ghana, Rwanda (introduced), Sierra Leone, South African Republic, Swaziland, Zambia. Middle East: Armenia, Azerbaijan, Georgia, Iran, Israel. North America: Canada, Mexico, USA. Central America: Costa Rica. Asia: Japan. Australasia: Australia, New Zealand.

Material examined: Israel. CM. Mt. Carmel National Park. On the ground. 7 February 2012. *Leg*. O. Godorova, *det*. M. Krakhmalnyi (HAI-G-105).

Previous records from Israel: Judean Mountains, Jerusalem. March 1953 (Kew). Upper Jordan Valley, Kinneret. 16 December 1955 (Kew) [49].

Notes: The species is characterized by the presence of a diaphragm separating the gleba from the sterile base, lack of peristome, and abundant septate paracapillitium. Some mycologists thought that these distinctive features were sufficient to place the species in the genus *Vascellum* [5,42]. However, subsequent molecular phylogenetic studies [2,41,43], as well as the present analysis, demonstrated that *V. pratense* groups with core species of the genus *Lycoperdon*, and thus should be treated as *L. pratense*.

Spores of the current specimen studied under SEM correspond well with SEM micrographs presented by Rimóczi [65], and have an ornamentation of columnar to conical processes with rounded or sharper apices, together with low thin anastomoses between processes.

## Figures and Tables

**Table 1:** Data on specimens involved in the phylogenetic study.

Taxonomic Name	Origin	Reference	Collection ID or Herbarium Number	Herbarium	GenBank Accession No.
*A. pyriforme* (Schaeff.) Vizzini = *Lycoperdon pyriforme* Schaeff.	Ukraine	Current study	HAI-G-007	HAI	OR594097
*A. pyriforme*	Israel	Current study	HAI-G-11	HAI	OR594096
*A. pyriforme*	Ukraine	Current study	HAI-G-71K	HAI	OR594095
*A. pyriforme*	Ukraine	Current study	HAI-G-92K	HAI	OR594094
*A. pyriforme*	Israel	Current study	HAI-G-159	HAI	OR594093
*Bovista aestivalis* (Bonord.) Demoulin	Israel	Current study	HAI-G-73	HAI	OR594145
*B. aestivalis*	Israel	Current study	HAI-G-103	HAI	OR594144
*B. aestivalis*	Sweden	[41]	M. Jeppson 1122	GB	DQ112620
*B. cretacea* T.C.E. Fr.	Norway	[41]	M. Jeppson 5702	GB	DQ112610
*B. dermoxantha* (Vittad.) De Toni	Germany	Current study	Dupl. no. 16 ex L6, # 4411 (as *Bovista. pusilla* (Batsch) Pers.)	LG	OR594143
*B. dermoxantha*	Sweden	[41]	M. Jeppson 4568 (as *Lycoperdon dermoxanthum*)	GB	DQ112579
*B. dermoxantha*	USA	[67]	Isolate NCP34	–	HQ235047
*B. dermoxantha*	USA	[67]	Isolate SCP2	–	HQ235050
*B. furfuracea* Pers.	Belgium	Current study	Dupl. no. 893 ex L6	LG	OR594142
*B. furfuracea*	Sweden	[41]	M. Jeppson 5435	GB	DQ112622
*B. graveolens* Schwalb	Sweden	[41]	Widgren 030816	GB	DQ112618
*B. limosa* Rostr.	Sweden	[47]	MJ060816	GB	EU915075
*B. nigrescens* Pers.	Sweden	[41]	M. Jeppson 7719	GB	DQ112612
*B. paludosa* Lév.	Germany	[26]	REG130882AB	REG	AJ237630
*B. plumbea* Pers.	Israel	Current study	HAI-G-8	HAI	OR594141
*B. plumbea*	Israel	Current study	HAI-G-24	HAI	OR594140
*B. plumbea*	Israel	Current study	HAI-G-172	HAI	OR594139
*B. plumbea*	Israel	Current study	HAI-G-218	HAI	OR594138
*B. plumbea*	Sweden	[41]	M. Jeppson 4856	GB	DQ112613
*B. polymorpha* (Vittad.) Kreisel	Germany	[26]	TENN58028	TENN	AJ237613
*B. promontorii* Kreisel	Spain	[41]	M. Jeppson 7770	GB	DQ112621
*B. pusilla* (Batsch) Pers.	Germany	[26]	TENN58031	TENN	AJ237631
*B. pusilla*	Norway	[47]	M. Jeppson 8305	GB	EU915072
*B. tomentosa* (Vittad.) De Toni (Submitted to GenBank as *Bovista minor*)	USA	[41]	Steinke 951015	GB	DQ112617
*Bovistella radicata* (Durieu & Mont.) Pat. = *Lycoperdon radicatum* Durieu & Mont.	USA	Current study	Dupl. no. 894 ex L6, # 5120	LG	OR594137
*B. radicata*	Germany	[26]	TENN58056	GFW	AJ237624
*Calvatia bicolor* (Lév.) Kreisel	USA	[43]	LMG756–58	The Gilbertson Mycological Herb. (ARIZ)	EU833651
*Calvatia candida* (Rostk.) Hollós	Israel	Current study	HAI-G-78	HAI	OR594136
*C. candida*	Hungary	[41]	M. Jeppson 3514	GB	DQ112624
*C. chilensis*?	Argentina	Unpublished	BAFC 26765 (as *Calvatia cyathiformis*)	–	AJ486966
*C. craniiformis* (Schwein.) Fr.	USA	Current study	Dupl. no. 895 ex L6, # 4124	LG	OR594135
*C. cretacea* (Berk.) Lloyd = *Lycoperdon cretaceum* Berk.	Norway	[41]	M. Jeppson 4302 (as *Lycoperdon cretaceum*)	GB	DQ112598
*C. cyathiformis* (Bosc) Morgan	France	Current study	Dupl. no. 896 ex L6, # 4530	LG	OR594134
*C. cyathiformis* var. *cyathiformis* (Bosc) Morgan	Paraguay	Unpublished	PC, 9-VI-1890	–	AJ486873
*C. fragilis* (Quél.) Morgan = *C. cyathiformis* (Bosc) Morgan?	France	[40]	GFW (Kreisel) leg. Lopez Nov. 1990	GFW	AJ617493
*C. gigantea* (Batsch) Lloyd = *Langermannia gigantea* (Batsch) Rostk.	Germany	[40]	CFRM FP-98552	–	AJ617492
*C. turneri* (Ellis & Everh.) Demoulin & M. Lange = *Lycoperdon turneri* Ellis & Everh	Greenland	[41]	Lange 08–95 (as *Lycoperdon turneri*)	–	DQ112596
*C. utriformis* (Bull.) Jaap = *Lycoperdon utriforme* Bull.	Belgium	Current study	Dupl. no. 21 ex L6, # 3545	LG	OR594133
*Disciseda bovista* (Klotzsch) Henn.	Sweden	[41]	M. Jeppson 5078	GB	DQ112627
*D. candida* (Schwein.) Lloyd	USA	[43,46]	STB 00304	–	EU833654
*Handkea excipuliformis* (Scop.) Kreisel = *Lycoperdon excipuliforme* (Scop.) Pers.	Ukraine	Current study	HAI-G-017	HAI	OR594132
*H. excipuliformis*	Ukraine	Current study	HAI-G-17K (as *Lycoperdon gemmatum* Batsch)	HAI	OR594131
*H. excipuliformis*	Sweden	[41]	M. Jeppson 6467 (as *Lycoperdon excipuliforme*)	GB	DQ112590
*H. fumosa* (Zeller) Kreisel = *Gastropila fumosa* (Zeller) P. Ponce de León	USA	[43,46]	STB 00776	–	EU833655
*H. subcretacea* (Zeller) Kreisel = *Lycoperdon subcretaceum* (Zeller) Jeppson & E. Larss.	USA	[43,46]	STB MGW543	–	EU833658
*Handkea utriformis* (Bull.) Kreisel = *Lycoperdon utriforme* Bull.	Sweden	[41]	M. Jeppson 5388 (as *Lycoperdon utriforme*)	GB	DQ112607
*H. utriformis*	USA	[43,46]	STB MGW541	–	EU833659
*Holocotylon brandegeeanum* Lloyd	USA	[43]	STB00111	–	EU833660
*Lepiota cristata* (Bolton) P. Kumm.	USA	[68]	Vellinga 2611	UC	AF391045
*Lycoperdon altimontanum* Kreisel	Nepal	[41]	Dobremez–holotypus	Herb. Kreisel	DQ112589
*L. atropurpureum* Vittad.	Israel	Current study	HAI-G-19	HAI	OR594130
*L. atropurpureum*	Israel	Current study	HAI-G-26	HAI	OR594129
*L. atropurpureum*	Israel	Current study	HAI-G-31	HAI	OR594128
*L. atropurpureum*	Israel	Current study	HAI-G-32	HAI	OR594127
*L. atropurpureum*	Israel	Current study	HAI-G-70	HAI	OR594126
*L. atropurpureum*	Israel	Current study	HAI-G-115	HAI	OR594125
*L. atropurpureum*	France	Current study	Dupl. no. 897 ex L6, # 3707	LG	OR594124
*L. atropurpureum* (Submitted to GenBank as *Lycoperdon* cf. *decipiens*)	Sweden	[41]	M. Jeppson 3269	GB	DQ112586
*L. caudatum* J. Schröt.	Sweden	[41]	R-G. Carlsson 920818	GB	DQ112633
*L. decipiens* Durieu & Mont.	Israel	Current study	HAI-G-63	HAI	OR594115
*L. decipiens*	Israel	Current study	HAI-G-75	HAI	OR594114
*L. decipiens*	Israel	Current study	HAI-G-142	HAI	OR594113
*L. decipiens*	Israel	Current study	HAI-G-145	HAI	OR594112
*L. decipiens*	Belgium	Current study	Dupl. no. 898 ex L6	LG	OR594111
*L. decipiens*	Sweden	[41]	M. Jeppson 7715	GB	DQ112583
*L.* cf. *dermoxanthum* Vittad.	USA	[43]	STB00624	–	FJ438478
*L. echinatum* Pers.	Belgium	Current study	Dupl. no. 566 ex L6	LG	OR594110
*L. echinatum*	Sweden	[41]	M. Jeppson 6498	GB	DQ112578
*L. ericaeum* Bonord.	Ukraine	Current study	HAI-G-004	HAI	OR594109
*L. ericaeum*	Sweden	[41]	M. Jeppson 4866	GB	DQ112606
*L. frigidum* Demoulin	USA	Current study	Dupl. no. 900 ex L6	LG	OR594108
*L. frigidum*	Svalbard (Norway)	[41]	Lange 191	–	DQ112559
*L. lambinonii* Demoulin	Belgium	Current study	Dupl. no. 515 ex L6, # 4349	LG	OR594107
*L. lambinonii*	Norway	[41]	M. Jeppson 5245	GB	DQ112576
*L.* cf. *lividum* Pers.	Belgium	Current study	Dupl. no. 34 ex L6, # 3530	LG	OR594117
*L.* cf. *lividum*	Israel	Current study	HAI-G-10	HAI	OR594123
*L.* cf. *lividum*	Israel	Current study	HAI-G-15	HAI	OR594122
*L.* cf. *lividum*	Israel	Current study	HAI-G-66	HAI	OR594121
*L.* cf. *lividum*	Israel	Current study	HAI-G-71	HAI	OR594120
*L.* cf. *lividum*	Israel	Current study	HAI-G-76	HAI	OR594119
*L.* cf. *lividum*	Israel	Current study	HAI-G-77	HAI	OR594118
*L. lividum* (Submitted to GenBank as *Lycoperdon niveum*)	Nepal	[41]	Dobremez 19740514	Herb. Kreisel	DQ112599
*L. mammiforme* Pers.	Sweden	[41]	M. Jeppson 4841	GB	DQ112567
*L. marginatum* Vittad.	USA	[43,46]	STB 00072	Herb. Bates	EU833661
*L.* cf. *molle* Pers.	Belgium	Current study	Dupl. no. 6 ex L6, # 3238	LG	OR594116
*L. molle*	Norway	[41]	M. Jeppson 4260	GB	DQ122566
*L. molle*	USA	[43]	STB 00098	Herb. Bates	EU833662
*L. muscorum* Morgan	Sweden	[15]	M. Jeppson 9017	GB	JN572905
*L. nigrescens* Pers.	Ukraine	Current study	HAI-G-008	HAI	OR594106
*L. nigrescens*	Ukraine	Current study	HAI-G-42K	HAI	OR594105
*L. nigrescens*	Ukraine	Current study	HAI-G-150K	HAI	OR594104
*L. nigrescens*	Sweden	[41]	M. Jeppson 5376	GB	DQ122577
*L.* cf. *niveum* Kreisel	Israel	Current study	HAI-G-72	HAI	OR594103
*L.* cf. *niveum*	Israel	Current study	HAI-G-74	HAI	OR594102
*L. niveum*	Iceland	[41]	M. Jeppson 4068	GB	DQ112571
*L. norvegicum* Demoulin	Sweden	[41]	M. Jeppson 5453	GB	DQ112631
*L. perlatum* Pers.	Israel	Current study	HAI-G-69	HAI	OR594101
*L. perlatum*	Israel	Current study	HAI-G-80	HAI	OR594100
*L. perlatum*	Israel	Current study	HAI-G-171	HAI	OR594099
*L. perlatum*	Israel	Current study	HAI-G-173	HAI	OR594098
*L. perlatum*	Sweden	[41]	M. Jeppson 4684	GB	DQ112630
*L. pratense* Pers. = *Vascellum pratense* (Pers.) Kreisel	Ukraine	Current study	HAI-G-88K	HAI	OR594089
*L. pratense*	Israel	Current study	HAI-G-105	HAI	OR594088
*L. pulcherrimum* Berk. & M.A. Curtis	USA	[43,46]	STB 00066	Herb. Bates	EU833663
*L. pusillum* Batsch = *Bovista pusilla* (Batsch) Pers.	Japan	[69]	Lp1 isolate (*L. pusillum*)	–	AB067724
*L. rimulatum* Peck	USA	[43,46]	STB 00112	Herb. Bates	EU833664
*L. rupicola* Jeppson, E. Larss. & M.P. Martín (Submitted to GenBank as *Lycoperdon* cf. “*ericeum*”)	Sweden	[15]	Vetter 407	GB	DQ112581
*L. subumbrinum* Jeppson & E. Larss.	Ukraine	Current study	HAI-G-010 (as *L*. cf. *molle* Pers.)		OR594091
*L. subumbrinum* (Submitted to GenBank as *Lycoperdon lambinonii*)	Sweden	[15]	M. Jeppson 6377 (Type)	GB	DQ112602
*L. umbrinum* Pers.	Luxembourg	Current study	Dupl. no. 834 ex L6	LG	OR594090
*L. umbrinum*	Sweden	[41]	M. Jeppson 4556	GB	DQ112591
*L. violascens* Cooke & Massee = *Calvatia violascens* (Cooke & Massee) R.T. Baker	Australia	Unpublished	K 77924 (Type)		AJ486967
*Lycoperdon* sp.	Israel	Current study	HAI-G-27	HAI	OR594092
*Morganella fuliginea* (Berk. & M.A. Curtis) Kreisel & Dring = *Lycoperdon fuligineum* Berk. & M.A. Curtis	Paraguay	[45]	TENN59070	TENN	AF485065
*M. pyriformis* (Schaeff.) Kreisel & D. Krüger = *Apioperdon pyriforme* (Schaeff.) Vizzini	Sweden	[41]	M. Jeppson 4849	GB	DQ112558
*M. subincarnata* (Peck) Kreisel & Dring = *Lycoperdon subincarnatum* Peck	Germany	[26]	REG106/81	–	AJ237626
*Mycenastrum corium* (Guers.) Desv.	Sweden	[41]	M. Jeppson 5467	GB	DQ112628
*Tulostoma kotlabae* Pouzar	Hungary	[41]	M. Jeppson 6623	GB	DQ112629
*V. pratense* = *Lycoperdon pratense* Pers. (Submitted to GenBank as *V*. cf. *intermedium*)	Czech Republic	[41]	M. Jeppson 5880	GB	DQ112556

In cases when specimen vouchers in GenBank and published studies do not match, vouchers provided in the original publications are presented.

**Table 2:** Species composition of the family Lycoperdaceae in Israel.

Genus	Species	Reference or Herbarium Number
*Apioperdon*	* *A. pyriforme* (Schaeff.) Vizzini	HAI-G-11
*Bovista*	* *B. aestivalis* (Bonord.) Demoulin	HAI-G-73
	*B. nigrescens* Pers.	[49]
	*B. plumbea* Pers.	[49]
	*B. pusilla* (Batsch) Pers.	[49] (as *Lycoperdon pusillum* Batsch)
*Calvatia*	* *C. candida* (Rostk.) Hollós	HAI-G-78
	*C. gigantea* (Batsch) Lloyd	[55] (as *Lycoperdon giganteum* Batsch)
*Disciseda*	*D. bovista* (Klotzsch) Henn.	[48,49] (as “*D. cervina* (Berk.) Hollós”)
*Lycoperdon*	*L. atropurpureum* Vittad.	[55]
	* *L. decipiens* Durieu & Mont.	HAI-G-145
	*L. lividum* Pers.	[49] (as *L. spadiceum* Schaeff.)
	*L. molle* Pers.	[55]
	* *L. niveum* Kreisel	HAI-G-72
	* *L. perlatum* Pers.: Pers.	HAI-G-80
	*L. pratense* Pers.	[49] (as *Vascellum pratense* (Pers.) Kreisel)

*—new additions to the Israeli mycobiota.

## Data Availability

The DNA sequence data that had been obtained during the current study were deposited at the GenBank database. The accession numbers are provided in Table 1 and Figure 2.
